# A novel gluconeogenic route enables efficient use of erythritol in zoonotic *Brucella*

**DOI:** 10.3389/fvets.2024.1328293

**Published:** 2024-03-27

**Authors:** Leticia Lázaro-Antón, Maria Veiga-da-Cunha, Aitor Elizalde-Bielsa, Nathalie Chevalier, Raquel Conde-Álvarez, Maite Iriarte, Jean Jacques Letesson, Ignacio Moriyón, Emile Van Schaftingen, Amaia Zúñiga-Ripa

**Affiliations:** ^1^Departamento de Microbiología y Parasitología – IDISNA, Universidad de Navarra, Pamplona, Spain; ^2^Groupe de Recherches Metaboliques, De Duve Institute, UCLouvain, Brussels, Belgium; ^3^Research Unit in Biology of Microorganisms (URBM), NARILIS, UNamur, Namur, Belgium

**Keywords:** *Brucella*, erythritol, gluconeogenesis, placenta, abortion

## Abstract

Brucellosis is a worldwide extended zoonosis caused by pathogens of the genus *Brucella*. While most *B. abortus*, *B. melitensis*, and *B. suis* biovars grow slowly in complex media, they multiply intensely in livestock genitals and placenta indicating high metabolic capacities. Mutant analyses *in vitro* and in infection models emphasize that erythritol (abundant in placenta and genitals) is a preferred substrate of brucellae, and suggest hexoses, pentoses, and gluconeogenic substrates use in host cells. While *Brucella* sugar and erythritol catabolic pathways are known, growth on 3–4 carbon substrates persists in Fbp- and GlpX-deleted mutants, the canonical gluconeogenic fructose 1,6-bisphosphate (F1,6bP) bisphosphatases. Exploiting the prototrophic and fast-growing properties of *B. suis* biovar 5, we show that gluconeogenesis requires fructose-bisphosphate aldolase (Fba); the existence of a novel broad substrate bisphosphatase (Bbp) active on sedoheptulose 1,7-bisphosphate (S1,7bP), F1,6bP, and other phosphorylated substrates; that *Brucella* Fbp unexpectedly acts on S1,7bP and F1,6bP; and that, while active in *B. abortus* and *B. melitensis*, GlpX is disabled in *B. suis* biovar 5. Thus, two Fba-dependent reactions (dihydroxyacetone-phosphate + glyceraldehyde 3-phosphate ⇌ F1,6bP; and dihydroxyacetone-phosphate + erythrose 4-phosphate ⇌ S1,7bP) can, respectively, yield fructose 6-phosphate and sedoheptulose 7-phosphate for classical gluconeogenesis and the Pentose Phosphate Shunt (PPS), the latter reaction opening a new gluconeogenic route. Since erythritol generates the PPS-intermediate erythrose 4-phosphate, and the Fba/Fbp-Bbp route predicts sedoheptulose 7-phosphate generation from erythrose 4-phosphate, we re-examined the erythritol connections with PPS. Growth on erythritol required transaldolase or the Fba/Fbp-Bbp pathway, strongly suggesting that Fba/Fbp-Bbp works as a PPS entry for both erythritol and gluconeogenic substrates in *Brucella*. We propose that, by increasing erythritol channeling into PPS through these peculiar routes, brucellae proliferate in livestock genitals and placenta in the high numbers that cause abortion and infertility, and make brucellosis highly contagious. These findings could be the basis for developing attenuated brucellosis vaccines safer in pregnant animals.

## Introduction

Members of the genus *Brucella* cause brucellosis, a highly contagious zoonosis severely affecting animal production and human health worldwide (1). Phylogenomic analyses show that most brucellae form a core group (the “classical” species *B. abortus, B. melitensis, B. suis, B. neotomae, B. ovis,* and *B. canis*, and their biovars, plus *B. pinnipedialis, B. ceti*, *B. papionis,* and *B. microti*) separated from early diverging brucellae and environmental *Ochrobactrum* species (2, 3).

**Figure 1:**
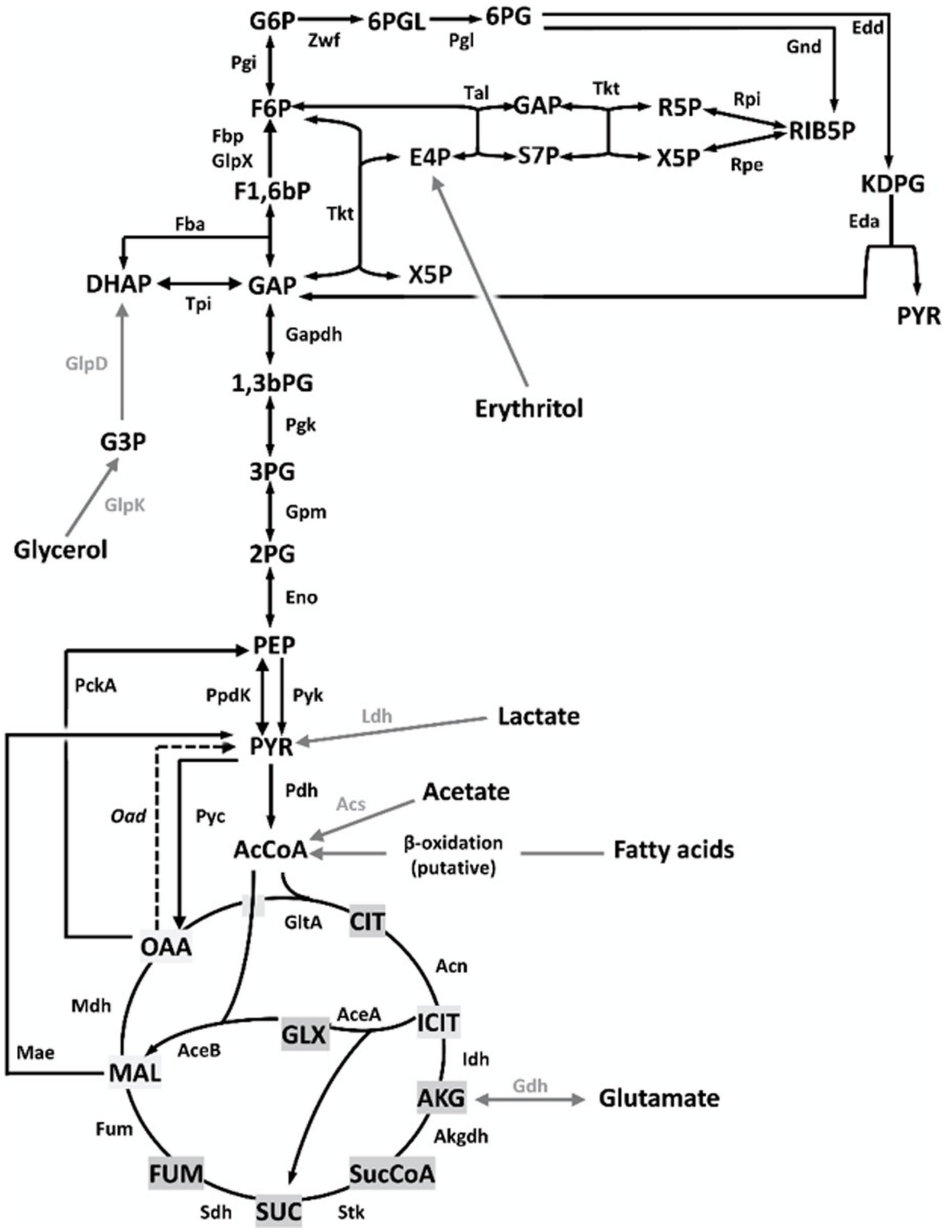
Central C metabolic network of *B. suis* 513 [adapted from Barbier et al. (18) and Zúñiga-Ripa et al. (12)]. The metabolic network includes complete Pentose Phosphate, Entner–Doudoroff, and gluconeogenesis pathways, as well as a complete tricarboxylic acid cycle including a glyoxylate shunt. Gray arrows and gray font indicate peripheral pathways. *Metabolites*: 1,3,bPG, 1,3 bisphosphoglycerate; KDPG, 2-keto-3-deoxy-phosphogluconate; 2PG, 2-phosphoglycerate; 3PG, 3-phosphoglycerate; 6PGL, 6-P-gluconolactone; 6PG, 6-phosphogluconate; AcCoA, acetyl-coenzyme A; AKG, alpha-ketoglutarate; CIT, citrate; ICIT, isocitrate; DHAP, dihydroxyacetone-P; E4P, erythrose 4-P; F1,6bP, fructose 1,6-bisphosphate; F6P, fructose 6-P; FUM, fumarate; G6P, glucose 6-P; GAP, glyceraldehyde 3-P; G3P, glycerol 3-P; GLX, glyoxylate; MAL, malate; OAA, oxaloacetate; PEP, phosphoenolpyruvate; PYR, pyruvate; R5P, ribose 5-P; RIB5P, ribulose 5-P; S7P, sedoheptulose 7-P; SUC, succinate; SucCoA, succinyl-coenzyme A; X5P, xylulose-5-P. *Enzymes*: Edd, 6-phospho-D-gluconate dehydratase; Gnd, 6-phosphogluconate dehydrogenase; Pgl, 6-phosphogluconolactonase; Acs, acetyl-coenzyme A synthetase; Acn, aconitate hydratase; Akgdh, alpha-ketoglutarate dehydrogenase; GltA, citrate synthase; Eno, enolase; Fbp/GlpX, fructose 1,6-bisphosphatase; Fba, fructose bisphosphate aldolase; Fum, fumarase; Zwf, glucose 6-P dehydrogenase; Pgi, glucose 6-P isomerase; Gdh, glutamate dehydrogenase; Gapdh, glyceraldehyde 3-P dehydrogenase; GlpD, glycerol 3-P dehydrogenase; GlpK, glycerol kinase; Idh, isocitrate dehydrogenase; AceA, isocitrate lyase; Eda, 2-dehydro-3-deoxy-phosphogluconate aldolase; Ldh, lactate dehydrogenase; Mdh, malate dehydrogenase; AceB, malate synthase; Mae, malic enzyme; PckA, phosphoenolpyruvate carboxykinase; Pgk, phosphoglycerate kinase; Gpm, phosphoglycerate mutase; Pyc, pyruvate carboxylase; Pdh, pyruvate dehydrogenase; Pyk, pyruvate kinase; PpdK, pyruvate phosphate dikinase; Rpi, ribose 5-P isomerase; Rpe, ribulose-5-P-3-epimerase; Sdh, succinate dehydrogenase; Stk, succinyl-coenzyme A synthethase; Tal, transaldolase; Tkt, transketolase; Tpi, triose P isomerase.

The best-known brucellae are facultative intracellular parasites able to establish long-lasting infections. While their ability to hinder innate immunity detection and to control intracellular trafficking are key traits of their pathogenicity (4–8), the metabolism underpinning their capacity to multiply in the hosts is imperfectly known. In livestock, these bacteria display a characteristic tropism for genitals and placenta, where their multiplication in high numbers (up to 10^14^ bacteria per conceptus) causes intense tissue damage leading to infertility, abortion, and a copious release of the pathogen. Such intense multiplication is thought to be linked to the use of erythritol, abundant in those tissues (9) and, as these brucellae do not multiply in the environment, it plays a key role in pathogen transmission. Moreover, studies in infection models suggest that brucellae multiply in host cells through the combined and/or sequential use of hexoses, pentoses, amino acids, and gluconeogenic substrates (10–14). While pathways for erythritol, hexose, and pentose metabolism are known (Figure 1), mutants simultaneously disabled in Fbp and GlpX (the only known fructose 1,6-bisphosphate bisphosphatases, [FBPases]) grow on 3- and 4-carbon substrates (12, 14). This is unexpected because these enzymes should be essential under these conditions as they catalyze the only irreversible gluconeogenic step linking 3 and 4 carbon precursors with glucose synthesis and the Pentose Phosphate Shunt (PPS) (Figure 1). Since glucose and the PPS provide indispensable precursors for envelope and nucleic acid synthesis, this observation suggests a new FBPase and/or gluconeogenic pathway in this intracellular parasite.

Described as fastidious because of their slow growth on the rich media used routinely and for primary isolation, most core brucellae grow slowly on relatively simple synthetic media (12–16), and *B. microti* and *B. suis* biovar 5 are comparatively fast-growing (12, 17). Specifically, *B. suis* biovar 5 grows fast on combinations of lactate, glycerol, and glutamate, thus showing broad biosynthetic abilities obviously including gluconeogenesis (12). Taking advantage of these characteristics of *B. suis* biovar 5, we first show that gluconeogenesis in these bacteria requires fructose-bisphosphate aldolase (Fba), an enzyme involved in the synthesis of fructose 1,6-bisphosphate (F1,6bP) and also able to catalyze sedoheptulose 1,7-bisphosphate (S1,7bP) generation. Then, we identified a broad-spectrum phosphatase (Bbp) active on F1,6bP, S1,7bP plus other phosphorylated polyols, and that *Brucella* Fbp (a classical fructose 1,6-bisphosphatase) is also active on these two bisphosphorylated substrates, while GlpX is inactive in *B. suis* biovar 5 (but not in *B. abortus* and *B. melitensis*). Accordingly, the *B. suis* biovar 5 model shows that two *Brucella* enzymes, Fbp and Bbp enable gluconeogenesis through both the classical fructose 6-phosphate pathway and sedoheptulose 7-phosphate, the latter being a new route. Significantly, the new route makes erythritol fueling into PPP very efficient, thus accounting for the intense multiplication of these bacteria in genital tissues, with the subsequent pathological effects.

## Materials and methods

### Bacterial strains and plasmids

These materials are listed in Supplementary Tables 1, 2, respectively. This study uses strains gifted from Instituto Nacional de Investigação Agrária e Veterinária (*B. suis* 513), Virginia Tech (*E. coli* JLD2402), Technische Universität Braunschweig (*E. coli* S17λpir), and Colorado State University (*E. coli* SM10λpir [pTNS2] and *E. coli* HB101 [pRK2013]). The University of Navarra did not require the study to be reviewed or approved by an ethics committee because the strains were a gift from the above-mentioned institutions. All brucellae were handled under BSL-3 containment.

### Culture conditions

*Brucella* strains were routinely grown in peptone-glucose broth (tryptic soy broth [TSB], Biomerieux) or in TSB-agar (TSA). When appropriate, the following antibiotics (all from Sigma) were used: kanamycin (Km; 50 μg/mL), ampicillin (Amp; 200 μg/mL), polymyxin (Pmx; 1.5 μg/mL), and/or chloramphenicol (Cm; 20 μg/mL). When needed, media were supplemented with 5% sucrose. The media used to study the phenotype of the metabolic mutants were peptone-glucose, Gerhardt’s defined medium (glutamate-lactate-glycerol) (16), or Plommet’s vitamin-mineral salts (15) modified by Barbier et al. (18) (Supplementary Table 3). BL21 cells were grown in Luria-Broth (LB) or in M9 minimal medium (Sigma) supplemented with glucose (1 mM), MgSO_4_ (1 mM) and CaCl_2_ (1 mM).

### Growth curves

To avoid carryover of media, the inocula were prepared as follows. First, bacteria were grown in 50 mL flasks containing 10 mL of peptone-glucose for 18 h at 37°C with orbital shaking. These exponentially growing bacteria were harvested (5 min at 13,000 × *g*), resuspended in 10 mL of fresh test medium at an OD_600_ of 0.1, and grown for 18 h at 37°C with orbital stirring. These preconditioned bacteria were harvested, resuspended at an OD_600_ of 0.1 in 1 mL of fresh test medium, dispensed as technical triplicates in multi-well plates (200 μL/well), incubated at 37°C in a Bioscreen C apparatus with continuous shaking, and the OD at 420–580 nm measured at 0.5 h intervals. All experiments were repeated at least three times. Controls with medium and no bacteria were included in all experiments.

### DNA manipulations

Genomic sequences were obtained from the National Centre for Biotechnology Information (NCBI) and Kyoto Encyclopedia of Genes and Genomes (KEGG) databases. Searches for DNA and protein homologies were performed using the NCBI BLAST tool (19). DNA sequencing was performed by the “Servicio de Secuenciación del Centro de Investigación Médica Aplicada” (CIMA, Universidad de Navarra, Pamplona, Spain). Sequence alignments were performed with Clustal Omega (20, 21). Restriction enzymes were used as recommended by the manufacturer. Primers were synthesized by Sigma. Plasmid and chromosomal DNA were extracted with the QIAprep Spin Miniprep and the QIAamp DNA Mini Kit (Qiagen), respectively. DNA was purified from agarose gels using the QIAquick Gel Extraction Kit (Qiagen).

### Mutagenesis and complementations

The *Bs5*Δ*fbp*Δ*glpX* construct was described previously (12, 14).

*Bs5*Δ*fba* deleted in *fba* (*B. suis* 513 homolog [genome not annotated] of *B. abortus* 2308W BAB2_0365) was obtained introducing suicide plasmid pAZI-38 (for the construction, see Supplementary Table 2) into *B. suis* 513 by conjugation (22), and its integration selected by resistance to Pmx and Km. Then, the loss of the plasmid causing either a deletion or a sibling revertant wild type was selected on 5% sucrose. The resulting colonies were screened by PCR with primers Fba-F1 and Fba-R4, which amplified a fragment of 559 bp in the mutants and 1,513 bp in the sibling revertants. The absence/presence of the deleted sequence in these two types of strains was verified using a primer (Fba-R5; 5’-GCTCACCTTCCACCGAAAT-3) hybridizing in the deleted region.

Mutant *Bs5*Δ*bbp* deleted in *bbp* (*B. suis* 513 homolog of BAB1_0448 in *B. abortus* 2308W) was constructed using plasmid pLLA-21 (see Supplementary Table 2), which was transformed into *E. coli* Stellar and subsequently into *E. coli* S17λpir. Then, it was transferred to *B. suis* 513 by conjugation. Plasmid integration was selected by Pmx and Km resistance, and the excisions generating the mutants or the sibling revertants by sucrose resistance. The resulting colonies were screened by PCR with primers Bbp-F1 and Bbp-R4, which amplified a fragment of 732 bp in the mutant strains and of 1,227 bp in the sibling revertants. The absence/presence of the deleted sequence was then verified using a primer (Bbp-R5; 5’-CCTGACTGCGCCCATTAT-3′) hybridizing in the region targeted for deletion.

To construct mutant *Bs5*Δ*fbp*Δ*glpX*Δ*bbp*, the mutator plasmid pLLA-21 was introduced into *Bs5*Δ*fbp*Δ*glpX* (12), after allelic exchange, the mutants and sibling revertants were selected as described above using primers Bbp-F1 and Bbp-R4, and the mutation was verified using primer Bbp-R5.

The mutants deleted in the transaldolase (*tal*, *B. suis* 513 homolog of BAB1_1813 in *B. abortus* 2308W) were *Bs5*Δ*tal* (*B. suis* 513 lacking nucleotides 25–597 of *tal*), *Bs5*Δ*fbp*Δ*glpX*Δ*tal* and *Bs5*Δ*fba*Δ*tal* (carrying the *tal* deletion in the *Bs5*Δ*fbp*Δ*glpX* and *Bs5*Δ*fba* backgrounds, respectively). *Bs5*Δ*tal* was obtained using the suicide plasmid pLLA-18 (see Supplementary Table 2), which was introduced into *Brucella* by conjugation. Upon selection of the integration by resistance to Pmx and Km, the plasmid loss causing either a deletion or a sibling revertant wild type phenotype was selected on 5% sucrose. The resulting colonies were screened by PCR with primers Tal-F1 and Tal-R4, which amplified a fragment of 707 bp in the mutants and of 1,280 bp in the sibling revertant strains. The absence/presence of the deleted sequence in these two types of strains was verified using a primer (Tal-R6, 5’-CGATAACGGCTGCTTCTTTC-3′) hybridizing in the region targeted for deletion. To construct *Bs5*Δ*fbp*Δ*glpX*Δ*tal* and *Bs5*Δ*fba*Δ*tal* the mutator plasmid pLLA-18 was introduced into *Bs5*Δ*fbp*Δ*glpX* and *Bs5*Δ*fba*, respectively. After allelic exchange, the mutants were selected as described above using primers Tal-F1 and Tal-R4, and the mutation was verified using primer Tal-R6.

Validity of all mutations was assessed by complementation (Supplementary Table 1 and Supplementary Figure 1).

### Construction of *fbp*, *bbp*, and *glpX* expression plasmids

To produce recombinant Fbp, a *B. suis* 513 *fbp*-carrying pET-21 plasmid (pLLA-25, see Supplementary Table 2) was constructed using the In-Fusion cloning technology (Clontech). The pLLA-25 expression plasmid was introduced and amplified in *E. coli* Stellar, sequenced, and finally transformed into BL21(DE3) competent *E. coli*. Positive clones were verified by PCR using primers Fbp-pET21-F and Fbp-pET21-R.

For Bbp, a *B. suis* 513 *bbp*-carrying pET-21 plasmid pLLA-26 (see Supplementary Table 2) was constructed. This expression plasmid was introduced into *E. coli* Stellar, sequenced and transformed into *E. coli* BL21(DE3) and positive clones were verified by PCR using primers Bbp-pET21-F and Bbp-pET21-R.

To obtain GlpX, a *B. suis* 513 *glpX*-carrying pET-21 plasmid (pLLA-24, see Supplementary Table 2) was constructed using the same In-Fusion cloning technology (Clontech). Yet, all expression attempts to produce recombinant GlpX failed (see Results). To solve this problem, the pLLA-24 sequence was modified by two sequential PCR site-directed mutagenesis to obtain plasmid pMVD-2 (see Supplementary Table 2). This plasmid allowed the expression of an active recombinant GlpX in which amino acids Asp139 and Pro316 were replaced by Gly139 and Arg316 (_Bs513_GlpX139Gly316Arg), a sequence identical to that found in *B. abortus* 2308W (see Results).

### Protein expression and purification

*E. coli* BL21(DE3) carrying the expression vectors were grown with aeration at 37°C in LB-Amp overnight. Using these cultures, two flasks with 200 mL of LB-Amp and two flasks with 200 mL of M9-Amp were inoculated (2 mL/flask) and incubated with stirring at 37°C until an OD_600_ of approximately 0.6. Then, they were cooled for 10 min in an ice bath and one flask of each medium was supplemented with IPTG (0.4 mM final concentration) to induce protein expression (the remaining two flasks were used as non-induced controls). To allow for protein expression, the bacteria were grown overnight at 30 or 18°C, after which they were recovered by centrifugation (7,000 × *g*, 10 min, 4°C) and the pellet was resuspended in 10 mL of lysis buffer containing 1 mg/mL lysozyme, 5 μg/mL antipain, 5 μg/mL leupeptin, and 300 mM NaCl in 50 mM Na phosphate buffer (pH 8). Bacteria were lysed by three cycles of freezing and thawing in liquid N_2_, the lysates were treated with DNAse I (125 units/mL), and the insoluble material was removed (40,000 × *g*, 40 min, 4°C). A 10 μL aliquot of the soluble fraction was used for expression analysis of the proteins by SDS-PAGE in 12–14% gradient gels and Page Blue Protein Staining (Thermo Scientific) and Western blotting. For the latter, a 6X-HisTag monoclonal (1/2000 dilution, 4°C, overnight) and a peroxidase-conjugated anti-mouse antibody (1/10000, 2.5 h at room temperature) (both from Thermofisher) were used. Blots were developed with peroxide solution and then with luminol enhancer solution (Thermofisher).

Lysates expressing the highest amount of each recombinant protein were used for protein purification. To this end, they were filtered (0.2 μm pore Vivaspin; Sartorius), diluted 1:3 in 20 mM imidazole, 300 mM NaCl, 50 mM sodium phosphate buffer (pH 7.4), and recombinant proteins purified by metal-affinity chromatography. To this end, samples were loaded onto a 1 mL HisTrap FF column (GE Healthcare) at a flow of 1 mL/min, the column was washed with 20 mM imidazole, 300 mM NaCl, 50 mM sodium phosphate buffer (pH 7.4), and proteins eluted with a 0–300 mM imidazole linear gradient in the same buffer. Fbp, GlpX, and Bbp were eluted in a single peak that was concentrated using a Vivaspin Turbo 15 device (Sartorius). Then, imidazole was removed by gel filtration using a PD-10 column (GE Healthcare) equilibrated with 25 mM HEPES (pH 7.4) containing 5 μg/mL antipain, 5 μg/mL leupeptin, 1 mM DTT, 100 mM NaCl, and 5 mM MgCl_2_, and proteins eluted in 5 mL of the same buffer were aliquoted and stored at −80°C until analysis. For measuring enzyme activity, stocks were diluted adequately in a buffer containing 25 mM HEPES (pH 7.4), 10% glycerol, 0.5 mg/mL BSA, and 1 mM DTT.

### Phosphatase quantitative colorimetric assay

Phosphatase activity was measured using a colorimetric assay for inorganic phosphate. The assay mixture (90 μL final volume) contained 25 mM HEPES (pH 7.4), 0.1 mg/mL BSA, 1 mM MgCl_2_, 0.1 mM EDTA, and 0.25 mM of the indicated substrate. The tubes were pre-warmed at 30°C and the reaction was started by adding the appropriate amount of Fbp (0.3 μg/mL; 6 μL of a 500-fold dilution of the stock of purified protein) or Bbp 0.6 μg/mL (6 μL of a 40-fold dilution of the stock of purified protein). After 10 min, the reaction was stopped by the addition of 10 μL of 2 M HCl and placing the tubes on ice for 10 min. After a 3 min centrifugation at top speed to remove precipitated material, 80 μL of the supernatant were dispensed into 96-flat bottom well microtiter plates to which 150 μL of a solution containing 1 vol. of 6.3% (NH_4_)_6_Mo_7_O_24_ (ammonium heptamolybdate) in 7.5 N HCl and 3 vols. of 0.3% malachite green were added (23). Blanks without addition of enzymes as well as a standard curve, with known concentrations of inorganic phosphate (0 to 8 nmol of KPi in 80 μL), were prepared and run in parallel in the same assay conditions. These were necessary for computing the enzymatic activities. The amount of enzyme added in the assay was previously tested in order to ensure the linearity of the reaction during the 10-min incubation at 30°C. Statistical significance was evaluated using One-sample *t*-test (*p < 0.05, **p < 0.01, ***p < 0.001, ****p < 0.0001).

### Synthesis of sedoheptulose 1,7-bisphosphate

Sedoheptulose 1,7-bisphosphate (S1,7bP) was synthesized in a mixture containing 5 mM erythrose 4-phosphate (E4P) and 5 mM dihydroxyacetone phosphate (DHAP) (both from Sigma) in 1 mM EDTA, 25 mM HEPES (pH 7.4). The reaction was started by addition of 5 μL of an 8.8 mg/mL rabbit muscle aldolase stock (Boehringer; controls contained no aldolase) and, after 60 min at 30°C, stopped with 100 μL of 60% perchloric acid followed by 100 μL of 3 M KHCO_3_, and denatured Fba removed by centrifugation (13,000 × *g* for 5 min at 4°C). Production of S1,7bP was followed by measuring the unreacted DHAP through NADH consumption in the reaction DHAP → glycerol-3-phosphate (G3P) catalyzed by rabbit muscle glycerol-3-phosphate dehydrogenase (Roche). To this end, 15 μL aliquots of the E4P-DHAP-EDTA-HEPES mixture were diluted in 0.8 mL of 0.15 mM NADH, 1 mM EDTA, 0.1 mg/mL BSA in 25 mM HEPES (pH 7.4), supplemented with glycerol-3-phosphate dehydrogenase (2 μL of 10 mg/mL stock), and NADH consumption at 30°C was measured at 340 nm in a Beckman Coulter DU 800 spectrophotometer using the mixture without E4P as the blank.

S1,7bP was purified on a 15 mL Dowex AG1-X8 ion exchange resin (200–400 mesh) column previously washed with 1 M NaCl and equilibrated in 25 mM HEPES (pH 7.4). The column was loaded with 6 mL of a 1:3 dilution in 10 mM HEPES of the reaction mixture and S1,7bP was eluted with a 240 mL-linear gradient of 0–1 M NaCl in 10 mM HEPES at a flow rate of 1 mL/min. Fractions (1 mL) containing S1,7bP (identified by colorimetric measurement of Pi; see below) were pooled, lyophilized, resuspended in 1 mM MgCl_2_, and desalted on Biogel P2 equilibrated in H_2_O.

### Fructose 1,6-bisphosphatase spectrophotometric assay

FBPase activity was assayed spectrophotometrically by coupling the production of F6P to NADH generation using yeast phosphoglucose isomerase (Pgi; F6P → glucose-6-P) and *Leuconostoc mesenteroides* glucose-6-phosphate dehydrogenase (G6PDH, Zwf; glucose 6-P + NAD → 6-P-gluconolactone + NADH). Desalted stock solutions of each of the two coupling enzymes (Pgi: 10 units/mg) and (G6PDH: 15 units/mg), both from Roche, were prepared by centrifuging (5 min at 18,000 × *g*; 4°C) 50 μL of each of the two enzyme suspensions, removing the clear supernatant and resuspending the protein pellet in 100 μL of 25 mM Hepes, 0.5 mg/mL BSA, and 1 mM DTT storing solution. FBPase activity for the three recombinant enzymes (_Bs513_GlpX139Gly316Arg, Bbp, and Fbp) was measured at 30°C in a reaction mixture (1 mL) containing the appropriate concentration of F1,6bP (0 to 0.5 mM), 0.5 mM NAD^+^, 0.5 mg/mL BSA, 1 mM MgCl_2_, 0.1 mM EDTA in 25 mM HEPES pH 7.4 and 1 μL of the desalted Pgi and G6PDH stock solutions. The reaction was started by adding the recombinant phosphatases and the generation of NADH was monitored at 340_nm_ at 30°C in a Beckman Coulter spectrophotometer DU 800. Metals (MgCl_2_ or ZnCl_2_) were tested by addition to the mixture before starting the reaction. Under these conditions, interference by any component of the mixture was ruled out. Statistical significance was evaluated using one-way ANOVA followed by Dunnett’s test (*p < 0.05, **p < 0.01, ***p < 0.001, ****p < 0.0001).

## Results

### Dysfunction of the putative Fba (fructose-bisphosphate aldolase) abrogates growth of *Brucella suis* 513 on 3 and/or 4 C substrates

**Figure 2:**
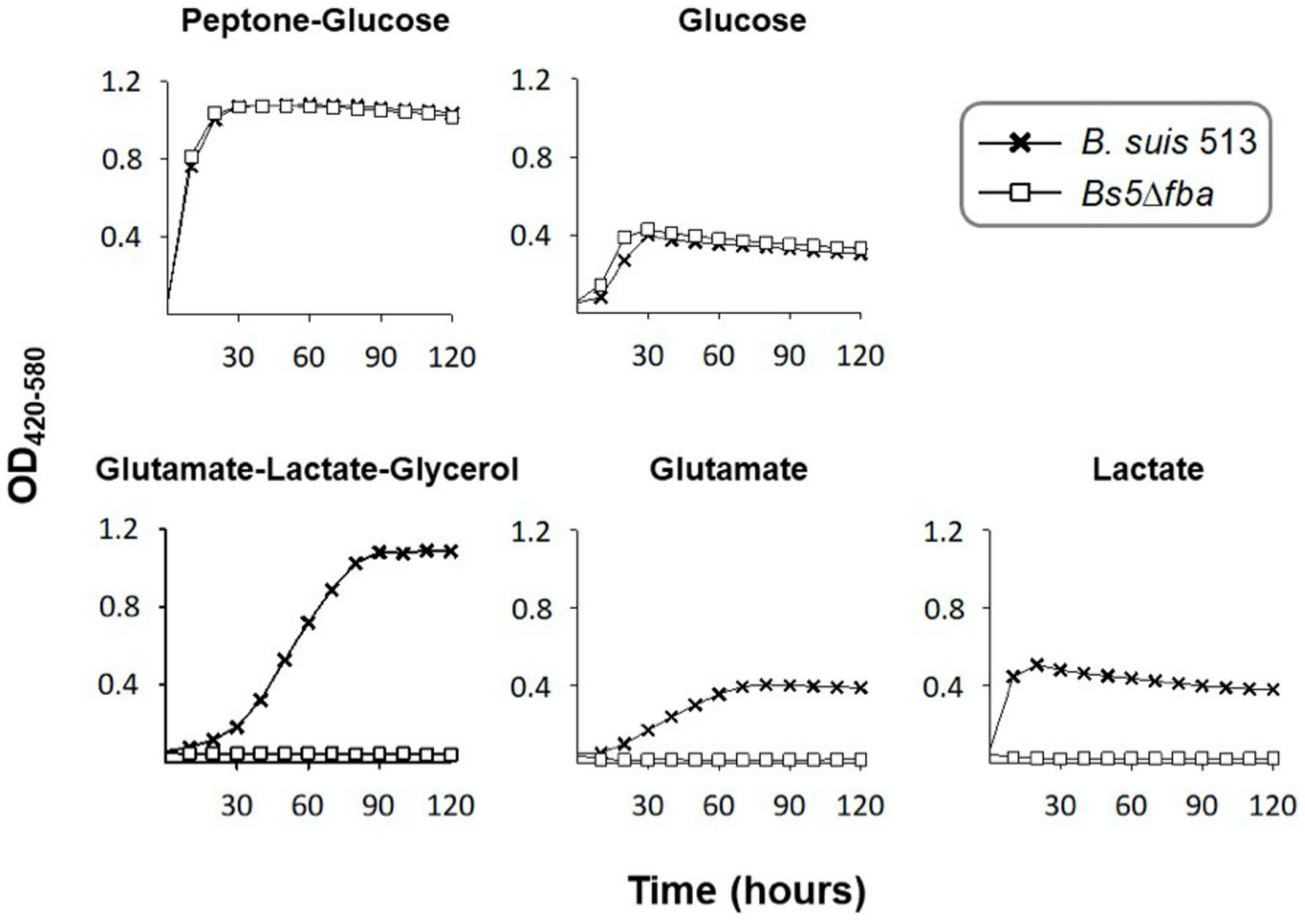
Dysfunction of the putative *fba* (*Bs5*Δ*fba* mutant) abrogates growth of *B. suis* 513 in gluconeogenic media. Each point represents the mean ± standard error (error bars are within the size of the symbols) of technical triplicates. The experiment was repeated three times with similar results.

*Brucella suis* 513 (biovar 5 reference strain) carries only one ORF putatively encoding a fructose-bisphosphate aldolase (Fba). The predicted enzyme belongs to the metal-dependent Class II Fbases, shows 88% sequence identity with *Rhizobium leguminosarum* bv. *viciae* Fba and conserves the aldolase activity domain (24). After making the appropriate mutant (*Bs5*Δ*fba*), we investigated whether the Fba-dependent dihydroxyacetone-phosphate (DHAP) + glyceraldehyde-3-phosphate (GAP) ⇌ F1,6bP interconversion (Figure 1) was affected by comparing growth on glucose or glucose-peptone and on 4 and 3 C substrates. Figure 2 shows that, while not affected in the former media, *Bs5*Δ*fba* did not grow on glutamate-lactate-glycerol, glutamate, or lactate, consistent with a role of this Fba in gluconeogenesis. Fba is highly conserved in other brucellae (99.44% identity with *B. abortus* 2308W and *B. melitensis* 16 M homologs).

### *Brucella* carries a putative phosphatase of the histidine superfamily

Considering this Fba requirement and the previously shown Fbp and GlpX dispensability for growth on 3 and 4 C substrates (12, 14), we investigated the existence of another FBPase. A perusal of the literature showed that a candidate bisphosphatase of the histidine superfamily had been described in *Saccharomyces* and *Mycobacterium* (25–27). The *B. suis* 513 genome contains two candidates belonging to this superfamily, the homologs of *B. abortus* 2308W BAB2_1013 and BAB1_0448 (*B. suis* 513 is not annotated). The KEGG annotation for BAB2_1013 is *gpm*, predicted to encode a protein with 49% identity with the human 2,3-bisphosphoglycerate-dependent phosphoglycerate mutase (Gpm; EC:5.4.2.11), and with this activity also assigned in BioCyc.^1^ Although BAB1_0448 is also annotated as gpm in KEGG, it has no specific role in BioCyc^2^, and the protein encoded shows only 22% identity with human Gpm. Considering this evidence and the results described below, we named the BAB1_0448 homolog *Brucella* broad-substrate phosphatase (*bbp*). *B. suis* 513 Bbp shows 99.49 and 100% identity with their *B. abortus* 2308W and *B. melitensis* 16 M homologs, respectively.

### The new phosphatase candidate (Bbp) is involved in gluconeogenesis in *Brucella*

**Figure 3:**
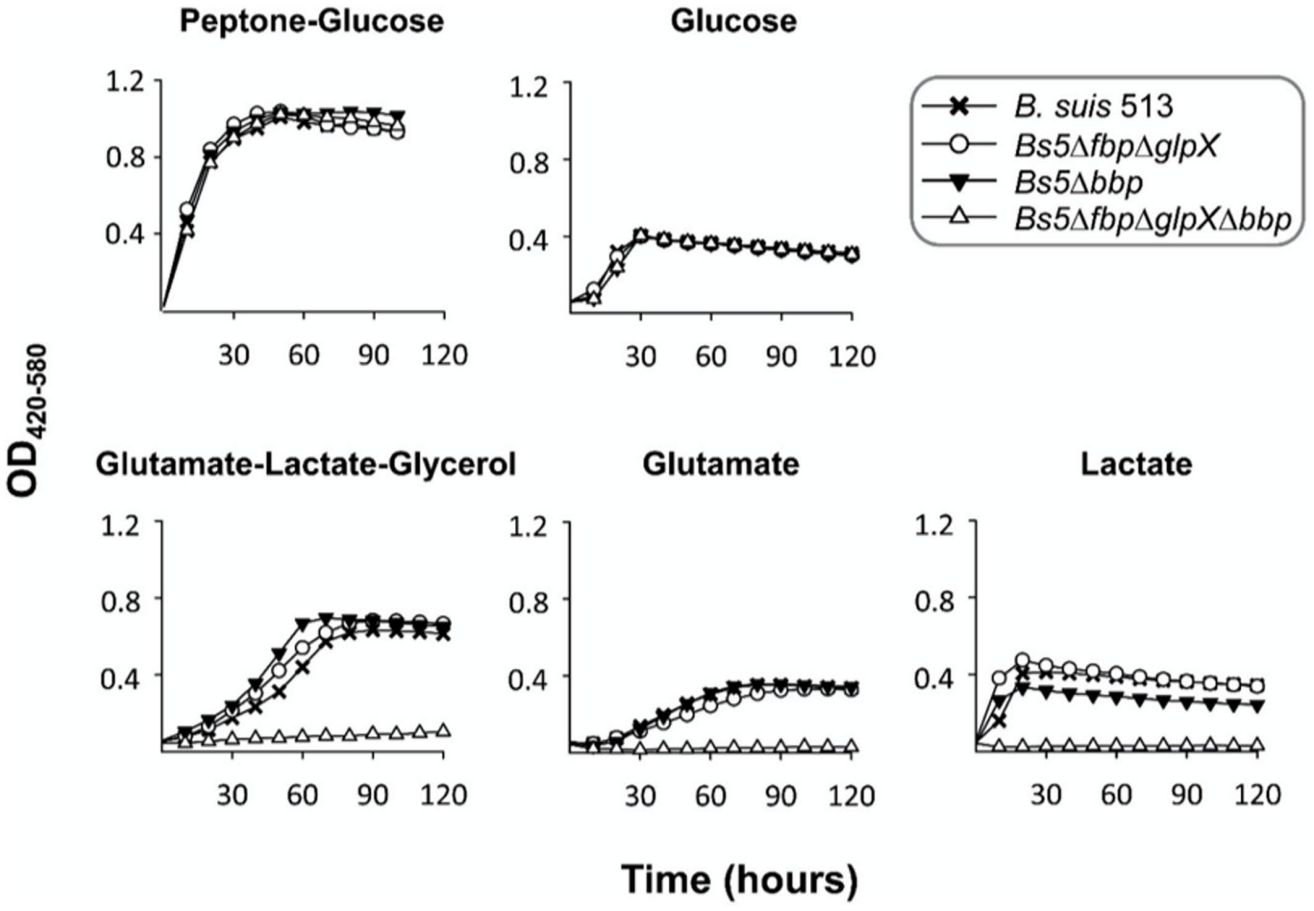
Dysfunction of *bbp* in *Bs5*Δ*fbp*Δ*glpX* abrogates growth in gluconeogenic media. Each point represents the mean ± standard error (error bars are within the size of the symbols) of technical triplicates. The experiment was repeated three times with similar results.

Figure 3 shows that *bbp* deletion alone (*Bs5*Δ*bbp* mutant) or combined with the canonical phosphatases (*Bs5*Δ*fbp*Δ*glpX*Δ*bbp* mutant) did not affect growth in non-gluconeogenic media (peptone-glucose and glucose). However, *Bs5*Δ*bbp* and *Bs5*Δ*fbp*Δ*glpX* but not *Bs5*Δ*fbp*Δ*glpX*Δ*bbp* grew under gluconeogenic conditions (Figure 3), proving that Bbp by itself can sustain gluconeogenesis. In parallel experiments, *B. abortus* Fbp, GlpX and Bbp, and *B. melitensis* Bbp restored growth on glycerol of Fbp- and GlpX- defective *E. coli*, thus confirming the gluconeogenic activity of *Brucella* Bbp (Supplementary Figure 2).

### *Brucella* Fbp, GlpX, and Bbp have fructose 1,6-bisphosphatase activity

To obtain *B. suis* 513 Fbp, GlpX, and Bbp, the corresponding open reading frames were cloned into a bacterial expression vector allowing for the overexpression in *E. coli* of the respective recombinant proteins. While this resulted in the expression of soluble Fbp and Bbp, GlpX appeared in insoluble inclusion bodies suggesting an inactive configuration. A close examination of the sequence revealed that *B. suis* 513 GlpX had only two amino acid changes with respect to the homologous protein in *B. abortus* 2308W. One of these changes, an Arg316Pro mutation, affects a highly conserved arginine that is predicted to be in the C-terminal ß-strand 8. Since proline is incompatible with a ß-strand, this mutation likely results in improper folding (Supplementary Figure 3). Furthermore, ß-strand 8 is largely responsible for the dimerization of GlpX that is required for the activity of the enzyme. On the other hand, the second mutation (Gly139Asp) is predicted to have only a minor effect as it is localized in a loop. These results strongly suggested that GlpX is not active in *B. suis* 513. To test this hypothesis, we used site-directed mutagenesis to replace Arg316 by proline and Asp139 by glycine in the GlpX expressing vector (henceforth named _Bs513_GlpX139Gly316Arg) obtaining a GlpX that was similar to that of *B. abortus* 2308W (whose activity was previously proved; Supplementary Figure 2). These changes resulted in the production of a soluble GlpX that was purified by Ni-affinity chromatography yielding an active protein of the expected molecular weight. Then, we studied the activity and kinetic properties of *B. suis* 513 Bbp and Fbp, and of _Bs513_GlpX139Gly316Arg. Table 1 shows that both Fbp and Bbp are able to catalyze the dephosphorylation of F1,6bP and S1,7bP with Kcat/Km values that did not markedly differ between the 2 enzymes. In contrast, GlpX preferred F1,6bP to S1,7bP as substrate.

**Table 1:** Kinetic properties and specificity of *Brucella* Bbp, Fbp, and GlpX^1^^,^^2^.

Enzyme	Substrate	Km (μM)	Vmax (μmol^−1^ min^−1^·mg^−1^)	Kcat (sec^−1^)	Kcat/Km (mM^−1^ s^−1^)
Bbp	F1,6bP	53.2 ± 6.3	13.9 ± 0.6	5.5	103
Fbp	F1,6bP	22.8 ± 2.0	6.7 ± 0.2	3.9	171
GlpX	F1,6bP	8.8 ± 1.1	1.1 ± 0.1	0.7	74
Bbp	S1,7bP	13.9 ± 3.2	11.3 ± 0.81	4.5	320
Fbp	S1,7bP	11.6 ± 1.9	14.3 ± 0.6	8.2	710
GlpX	S1,7bP	36.8 ± 5.6	0.6 ± 0.1	0.3	9

1 Values are the mean ± standard error of three technical replicates. Three biological replicates performed did not differ by more than 10%. The activities were measured with F1,6bP (spectrophotometric assay) or S1,7bP (Itaya assay) as substrate.

2 *B. suis* GlpX modified to correct inactivating mutations according to the *B. abortus* 2308W sequence (i.e., _Bs513_GlpX139Gly316Arg).

### Bbp is a metal-independent broad-range phosphatase

**Figure 4:**
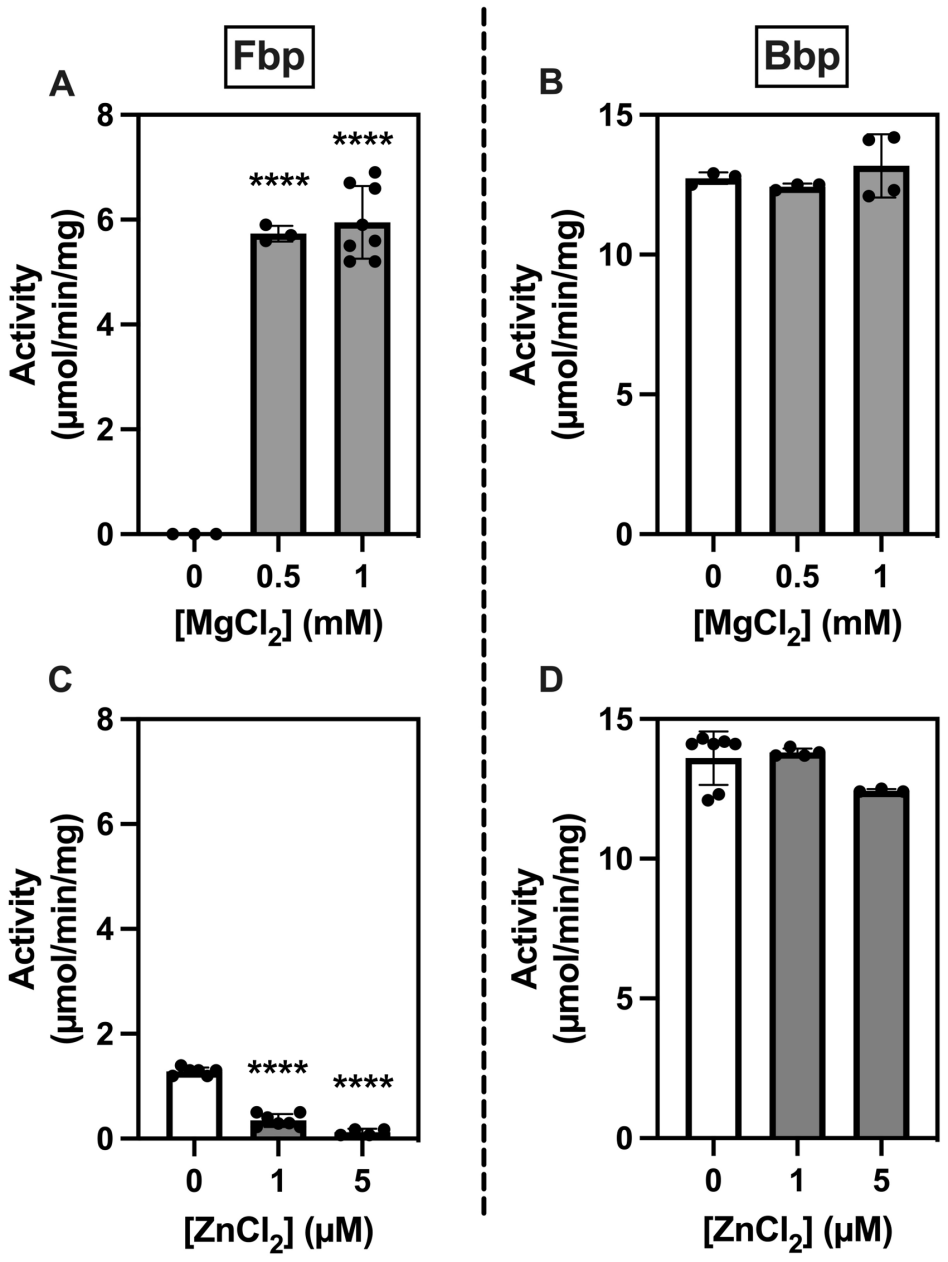
*Brucella* Fbp requires Mg^2+^ for activity and is inhibited by Zn^2+^ while Bbp does neither require Mg^2+^ for activity nor is inhibited by Zn^2+^. Fbp **(A,C)** and Bbp **(B,D)** phosphatase activity was measured in a spectrophotometric assay in the presence of 0.5 mM F1,6bP as substrate and the indicated concentration of metals. The measurements were repeated three times. Statistical significance was evaluated using one-way ANOVA followed by Dunnett’s test (*p < 0.05, **p < 0.01, ***p < 0.001, ****p < 0.0001).

Since members of the histidine phosphatase superfamily differ from other phosphatases in metal requirements and sensitivity to inhibition by metal ions (25), we examined the effects of MgCl_2_ and ZnCl_2_ on the activity of Fbp and Bbp on F1,6bP using a spectrophotometric assay. Phosphatase activity of Fbp but not of Bbp required Mg^2+^ (Figures 4A,B). Conversely, Fbp but not Bbp activity was inhibited by Zn^2+^ (Figures 4C,D). When testing the inhibition of Fbp activity by Zn^2+^ (Figure 4C), although 1 mM MgCl_2_ was added it was not possible to add 0.1 mM EDTA (as in Figure 4A) because it prevents the effect of Zn^2+^. This explains the 6-fold lower activity seen for Fbp in these conditions (Figure 4C).

**Figure 5:**
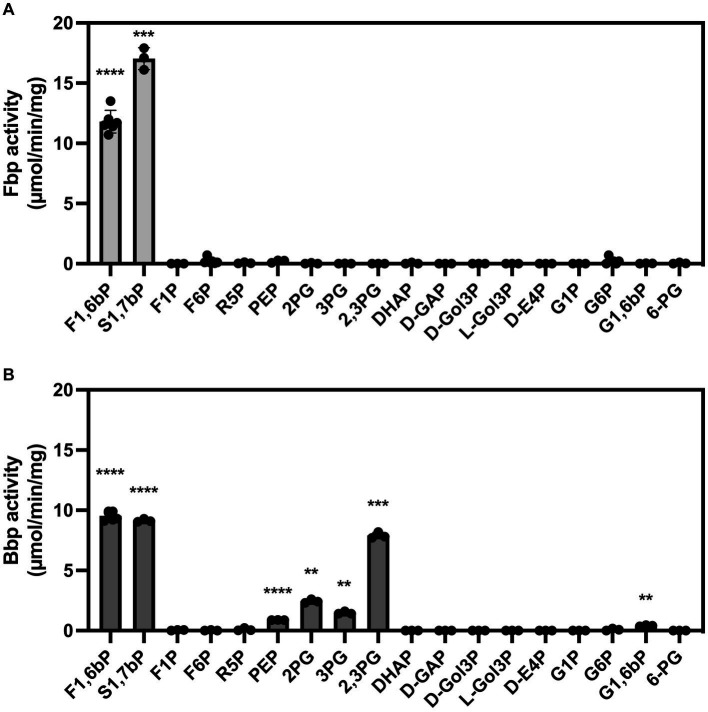
Substrate specificity of *B. suis* 513 Fbp **(A)** and Bbp **(B)**. The phosphatase activity was measured in the presence of 0.25 mM of the indicated substrates using a colorimetric assay that quantifies the inorganic phosphate formed during the reaction. The measurements were repeated three times. F1,6bP, fructose 1,6-bisphosphate; S1,7bP, sedoheptulose 1,7-bisphosphate; F1P, fructose 1-phosphate; F6P, fructose 6-phosphate; R5P, ribose 5-phosphate; PEP, phosphoenolpyruvate phosphate; 2PG, 2-phosphoglycerate; 3PG, 3-phosphoglycerate; 2,3PG, 2,3-bisphosphoglycerate; DHAP, dihydroxyacetone-phosphate; D-GAP, D-glyceraldehyde-3-phosphate; D-Gol3P, D-3-phosphoglycerol; L-Gol3P, L-3-phosphoglycerol; D-E4P, D-erythrose 4-phosphate; G1P, glucose 1-phosphate; G6P, glucose 6-phosphate; G1,6bP, glucose 1,6-bisphosphate; 6-PG, 6-phosphogluconate. Statistical significance was evaluated using One-sample *t*-test (*p < 0.05, **p < 0.01, ***p < 0.001, ****p < 0.0001).

Concerning substrate specificity, Fbp dephosphorylated F1,6bP and S1,7bP but did not dephosphorylate the other substrates tested (Figure 5A and Table 1). In contrast, Bbp acted on F1,6bP and S1,7bP but also on 2,3-phosphoglycerate (2,3PG) and at lower rates on 2PG, 3PG, PEP, and G1,6bP (Figure 5B). Altogether, Bbp is a less specific bisphosphatase than Fbp.

### Erythritol bypasses Fba-mediated gluconeogenesis in *Brucella suis* 513

**Figure 6:**
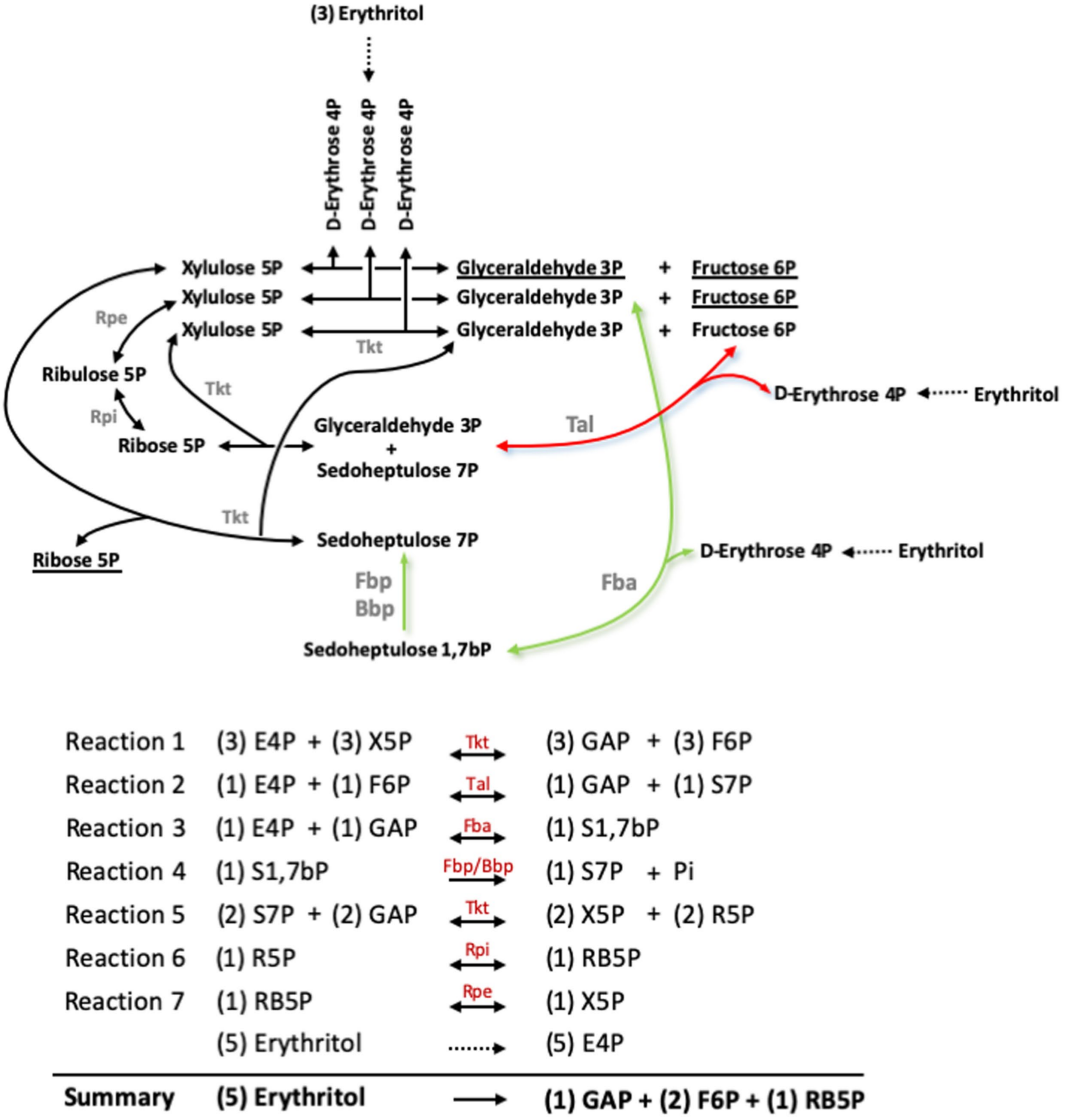
Pathway proposed for erythritol catabolism through the Pentose Phosphate reactions and the sedoheptulose 1,7-bP bypass. Red arrows indicate the Tal-mediated pathway proposed by Barbier et al. (27), and green arrows indicate the Fba-mediated pathway. Underlined substrates indicate the net production of the pathway. GAP, glyceraldehyde 3-phosphate; E4P, erythrose 4-phosphate; F6P, fructose 6-phosphate; R5P, ribose 5-phosphate; RB5P, ribulose 5-phosphate; S1,7bP, sedoheptulose 1,7-bisphosphate; S7P, sedoheptulose 7-phosphate; X5P, xylulose 5-phosphate.

**Figure 7:**
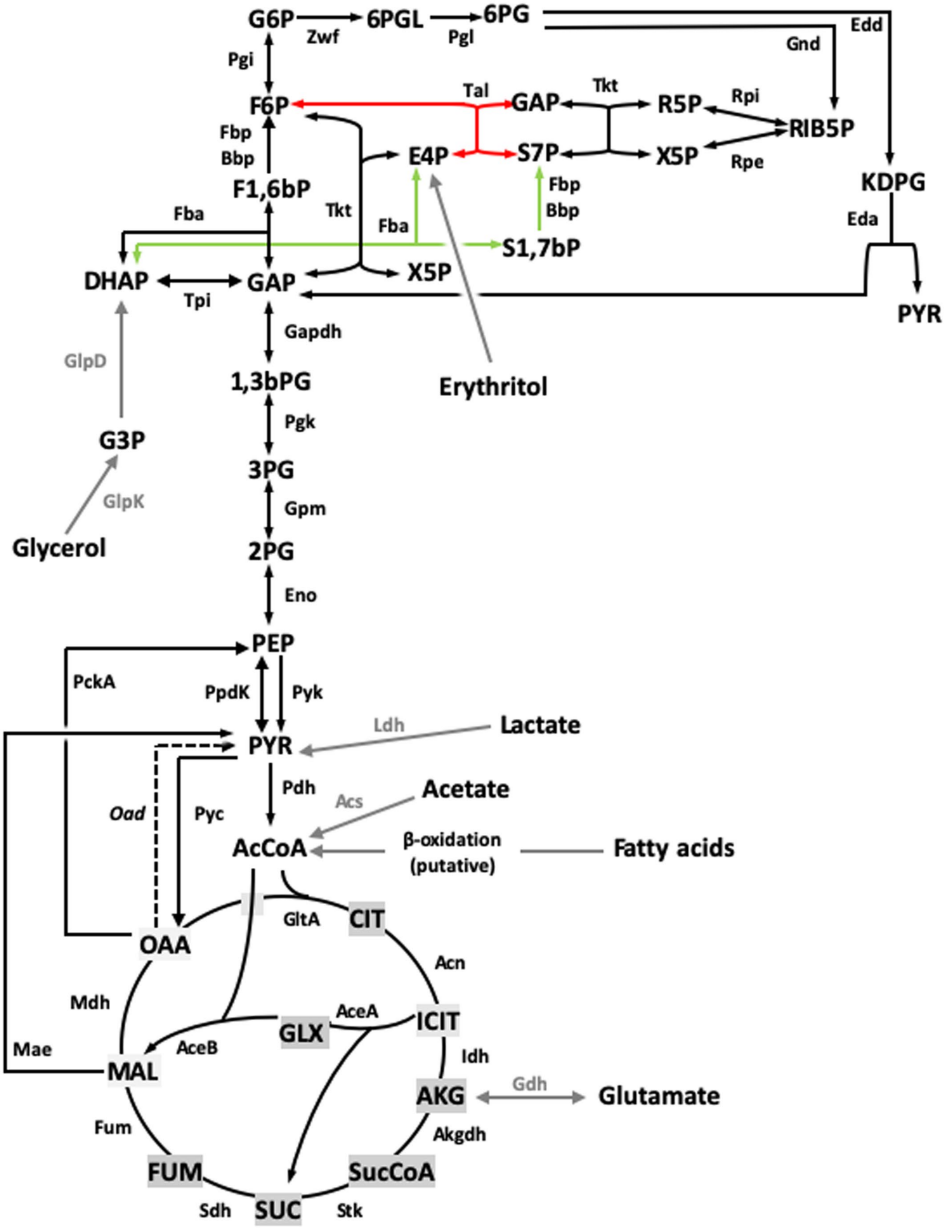
Revised model for the Central C metabolic network of *B. suis* 513 [adapted from Barbier et al. (18) and Zúñiga-Ripa et al. (12)]. The metabolic network includes complete Pentose Phosphate, Entner–Doudoroff, and gluconeogenesis pathways, as well as a complete tricarboxylic acid cycle including a glyoxylate shunt. This revised version of the metabolic network includes the sedoheptulose 1,7-bisphosphate bypass described in this work. Regarding the last steps of erythritol catabolism, red and green arrows indicate the Tal- and the Fba- mediated pathways, respectively. Gray arrows and gray font indicate peripheral pathways. *Metabolites*: 1,3,bPG, 1,3 bisphosphoglycerate; KDPG, 2-keto-3-deoxy-phosphogluconate; 2PG, 2-phosphoglycerate; 3PG, 3-phosphoglycerate; 6PGL, 6-P-gluconolactone; 6PG, 6-phosphogluconate; AcCoA, acetyl-coenzyme A; AKG, alpha-ketoglutarate; CIT, citrate; ICIT, isocitrate; DHAP, dihydroxyacetone-P; E4P, erythrose 4-P; F1,6bP, fructose 1,6-bisphosphate; F6P, fructose 6-P; FUM, fumarate; G6P, glucose 6-P; GAP, glyceraldehyde 3-P; G3P, glycerol 3-P; GLX, glyoxylate; MAL, malate; OAA, oxaloacetate; PEP, phosphoenolpyruvate; PYR, pyruvate; R5P, ribose 5-P; RIB5P, ribulose 5-P; S1,7bP, sedoheptulose 1,7-bisphosphate; S7P, sedoheptulose 7-P; SUC, succinate; SucCoA, succinyl-coenzyme A; X5P, xylulose-5-P. *Enzymes*: Edd, 6-phospho-D-gluconate dehydratase; Gnd, 6-phosphogluconate dehydrogenase; Pgl, 6-phosphogluconolactonase; Acs, acetyl-coenzyme A synthetase; Acn, aconitate hydratase; Akgdh, alpha-ketoglutarate dehydrogenase; Bbp, *Brucella* broad phosphatase; GltA, citrate synthase; Eno, enolase; Fbp, fructose 1,6-bisphosphatase; Fba, fructose bisphosphate aldolase; Fum, fumarase; Zwf, glucose 6-P dehydrogenase; Pgi, glucose 6-P isomerase; Gdh, glutamate dehydrogenase; Gapdh, glyceraldehyde 3-P dehydrogenase; GlpD, glycerol 3-P dehydrogenase; GlpK, glycerol kinase; Idh, isocitrate dehydrogenase; AceA, isocitrate lyase; Eda, 2-dehydro-3-deoxy-phosphogluconate aldolase; Ldh, lactate dehydrogenase; Mdh, malate dehydrogenase; AceB, malate synthase; Mae, malic enzyme; PckA, phosphoenolpyruvate carboxykinase; Pgk, phosphoglycerate kinase; Gpm, phosphoglycerate mutase; Pyc, pyruvate carboxylase; Pdh, pyruvate dehydrogenase; Pyk, pyruvate kinase; PpdK, pyruvate phosphate dikinase; Rpi, ribose 5-P isomerase; Rpe, ribulose-5-P-3-epimerase; Sdh, succinate dehydrogenase; Stk, succinyl-coenzyme A synthethase; Tal, transaldolase; Tkt, transketolase; Tpi, triose P isomerase.

Barbier et al. (18) showed that erythritol is catabolized into erythrose 4-phosphate (E4P), and proposed that metabolism proceeds in the PPS through a transaldolase (Tal) and two transketolase (Tkt) catalyzed reactions (Figures 6, 7, red arrow). However, the new Fba/Fbp-Bbp pathway can produce sedoheptulose 7-phosphate (S7P) from E4P, suggesting a new entry into PPS and a third Tkt-dependent reaction, as depicted in the model in Figures 6, 7 (green arrows).

**Figure 8:**
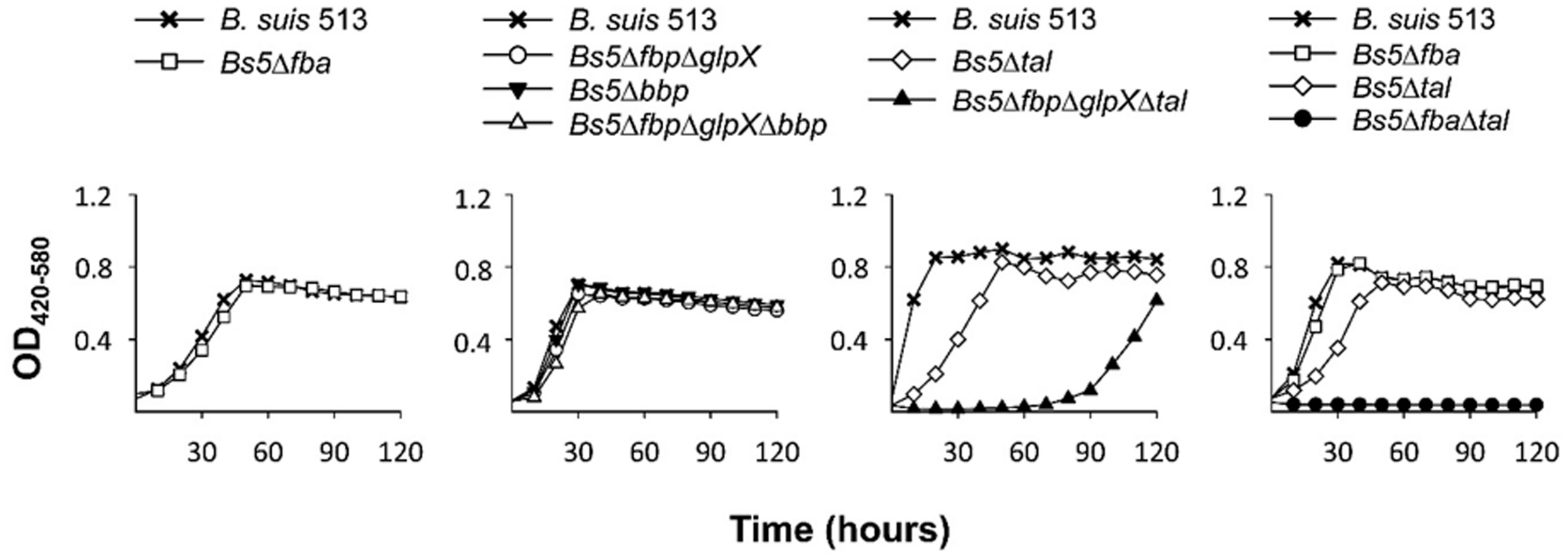
Growth of *B. suis* 513 on erythritol does not proceed in the absence of the SBPase and the Tal-dependent pathways. Each point represents the mean ± standard error (error bars are within the size of the symbols) of technical triplicates. The experiment was repeated three times with similar results.

Both this model and the one proposed by Barbier et al. (18) predict that erythritol can maintain growth through PPS via the Tal-mediated reaction (Figures 6, 7, red arrow) without using the Fba/Fbp-Bbp pathway. We confirmed this using *Bs5*Δ*fba* (Figure 8A), *Bs5*Δ*bbp*, *Bs5*Δ*fbp*Δ*glpX,* and *Bs5*Δ*fbp*Δ*glpX*Δ*bbp* (Figure 8B) (we included the *glpX* deletion to make sure that no residual activity of the mutated enzyme interfered in the results). Another prediction of the new model is that a *Bs5*Δ*tal* mutant should grow on erythritol only if the proposed Fba/Fbp-Bbp SBPase bypass (Figures 6, 7, green arrows) is active, and we observed this (Figure 8C). Interestingly, the *Bs5*Δ*tal* growth curves showed a lag phase not detected in the SBPase bypass mutants under the same conditions. Fully consistent with the role of Bbp in this SBPase bypass, a *Bs5*Δ*fbp*Δ*glpX*Δ*tal* mutant was able to grow on erythritol (Figure 8C). Although exponential phase generation times and final yields were not strikingly different from those of *Bs5*Δ*tal*, the *Bs5*Δ*fbp*Δ*glpX*Δ*tal* mutant showed a markedly long lag phase (Figure 8C). This result suggests that both Fbp and Bbp are necessary for full efficiency of the SBPase pathway in the presence of erythritol and, since the activities *in vitro* of the purified Fbp and Bbp were not strikingly different, that there is a delayed expression of *bbp* or other metabolic adjustments under these conditions. Finally, as proof that the Fba/Fbp-Bbp- and Tal-dependent pathways are the only ones involved, we demonstrated that a *Bs5*Δ*fba*Δ*tal* failed to grow on erythritol (Figure 8D).

## Discussion

As expected from a fructose-bisphosphate aldolase involved in the trioses-P ⇌ F1,6bP interconversion and in agreement with the homology analyses, we found that the putative aldolase gene (*fba*) is required for growth on gluconeogenic substrates, supporting that gluconeogenesis in *B. suis* 513 requires the action of F1,6bP bisphosphatases. Then, we confirmed the dispensability of Fbp and GlpX for growth on gluconeogenic substrates and found that the genomes of *B. suis* 513, *B. abortus* 2308W, and *B. melitensis* 16 M carry a gene coding for a broad substrate phosphatase (Bbp). Concerning *glpX*, we found that it codes for a non-functional enzyme in *B. suis* 513 which, however, is active in at least *B. abortus*. These differences may indicate the dispensability of GlpX in this pathogen but, while they do not rule out a contribution of GlpX in other brucellae, the results show that *fbp* and *bbp* (together or individually) can sustain growth on gluconeogenic substrates. Consistent with this, proof of their functionality was also provided by enzymatic analyses.

Bbp does not belong to any of the five groups of FBPases known (28), differing in structure, metal requirements, sensitivity to inhibitors, and substrate specificity. Remarkably, Bbp was active on S1,7bP, F1,6bP, and other bisphosphate sugars, and *Brucella* Fbp was active on both F1,6bP and S1,7bP. Their FBPase activity supports that *B. suis* 513 Fba and Fbp-Bbp can generate F6P from trioses via the sequential activities of the classical gluconeogenesis (i.e., DHAP + GAP ⇌ F1,6bP, and F1,6bP → F6P + Pi; Figure 1), which can explain the phenotypes observed for the Fbp and Bbp mutants. However, their SBPase activity is intriguing. In bacteria, this activity has been described in the facultative H_2_-chemolitotroph *Cupriavidus necator* H16 (formerly *Alcaligenes eutrophus* H16), where it accomplishes an essential role in the Calvin cycle (29). Also, a dual FBPase/SBPase activity was described for the plasmid-encoded GlpX^P^ of *Bacillus methanolicus* (28). In combination with Fba, this class II FBPase plays a role in the methylotrophic ribulose-phosphate pathway (classical FBPase activity) and in a new variant in which S1,7bP is dephosphorylated to S7P. None of these pathways is related to gluconeogenesis but the dual FBPases/SBPases could be related to a metabolic adaptation of brucellae facilitating the use of erythritol.

In *B. suis* 513 and absence of erythritol, in addition to the classical pathway (GA3P ⇌ DHAP; DHAP + GAP ⇌ F1,6bP; F1,6bP → F6P + Pi), gluconeogenesis produces F6P through the following three reactions: DHAP + E4P ⇌ S1,7bP (catalyzed by Fba); S1,7bP + H_2_O → S7P + Pi (catalyzed by Fbp and Bbp); S7P + GAP ⇌ F6P + E4P (catalyzed by Tal). While F6P is produced from DHAP and GAP, it is important to note that E4P plays a catalytic role in this set of reactions, and therefore it must be formed in some way to initiate the bypass. When the bacteria are growing on gluconeogenic substrates (and no erythritol), E4P is necessarily produced via the transketolase of PPS (F6P + GAP ⇌ xylulose 5-phosphate + E4P). This implies that some F6P needs to be first formed and, as F1,6bP (from DHAP and GAP) is the only possible source of F6P under these growth conditions, FBPase activity is necessary and a strictly specific SBPase would not be able to support growth *in vitro* of the Fbp mutant of *B. suis* 513 (where GlpX is inactive) on gluconeogenic substrates. Therefore, a dual role for Bbp is necessary, consistent with the hypothesis that the bypass is functional. Remarkably, there is another source of E4P not connected to Fba that is very relevant in *Brucella* pathogenesis. Elucidation of the full pathway for erythritol catabolism in these bacteria has shown to yield only E4P, and not DHAP as previously thought (18). Noteworthy, erythritol stimulates *Brucella* growth in minute amounts (0.4-4 μg/ml in McCullough and Dick’s media), an effect that has remained unexplained since first observed (30) and that could be explained by the catalytic role of E4P discussed above. Moreover, it has been proposed that, coupled to the erythritol pathway, synthesis of F6P and GAP through PPS occurs (18), which could be coupled to both gluconeogenesis and the reactions leading to the Krebs cycle. However, if erythritol is abundant, high amounts of E4P would be formed, which could make significant the production of S1,7bP by Fba and, therefore, the SBPase bypass a relevant way to increase the use of the abundant erythritol in the genitals and placenta of natural hosts.

While *B. suis* biovar 5 is more prototrophic than *B. abortus* 2308W, *B. melitensis* 16 M, or the slow-growing *B. suis* biovars (31), sequence analyses show that *fba*, *fbp,* and *bbp* are highly conserved, and at least *B. abortus* and *B. melitensis* Bbp show activity in gluconeogenic-deficient *E. coli*, strongly suggesting that the gluconeogenic pathways proposed here are functional in those species. This inference and the connection with erythritol metabolism in other brucellae are being currently verified.

As indicated in the Introduction, the brucellae can reach exceedingly high numbers in the placenta. Reaching such high numbers necessarily requires a very active and efficient use of available substrates, among which erythritol is conspicuous. Therefore, the intense multiplication and tissue damage, subsequent abortion and infertility, and pathogen release in high numbers are characteristics of brucellosis in all likelihood linked to the peculiar metabolic characteristic described here.

As with infection with field strains, the most effective vaccines against brucellosis—*B. abortus* S19 for cattle and *B. melitensis* Rev1 for small ruminants—induce abortion, an undesirable effect that hinders their use in mass vaccination campaigns. A possible strategy to avoid or reduce this abortifacient effect is to suppress their genital tropism. Since disrupting the use of erythritol might reduce the bacterial load in the placenta and subsequent reproductive failure, the results of this work provide the basis for the development of safer vaccines. Also, an improved understanding of bacterial metabolism offers opportunities for exploring new therapeutic agents.

## Data availability statement

The original contributions presented in the study are included in the article/Supplementary material, further inquiries can be directed to the corresponding author.

## Author contributions

LL-A: Conceptualization, Investigation, Methodology, Validation, Visualization, Writing – original draft, Writing – review & editing. MV-d-C: Investigation, Methodology, Supervision, Validation, Writing – review & editing. AE-B: Investigation, Writing – review & editing. NC: Investigation, Writing – review & editing. RC-Á: Conceptualization, Funding acquisition, Resources, Supervision, Writing – review & editing. MI: Investigation, Writing – review & editing. JL: Conceptualization, Writing – review & editing. IM: Conceptualization, Funding acquisition, Project administration, Supervision, Validation, Visualization, Writing – original draft, Writing – review & editing. ES: Conceptualization, Supervision, Visualization, Writing – review & editing. AZ-R: Conceptualization, Investigation, Methodology, Project administration, Supervision, Validation, Visualization, Writing – original draft, Writing – review & editing.

## Footnotes

^1^ https://biocyc.org/gene?orgid=GCF_000054005&id=BAB_RS31110#

^2^ https://biocyc.org/gene?orgid=GCF_000054005&id=BAB_RS18055
