# Supplementary material for: A novel gluconeogenic route enables efficient use of erythritol in zoonotic Brucella
Source: Front Vet Sci. 2024 Mar 27;11:1328293. doi: 10.3389/fvets.2024.1328293 (PMC11005471; doi:10.3389/fvets.2024.1328293)
Supplement: Supplementary file 1 [file Data_Sheet_1.PDF]

Supplementary Material

A novel gluconeogenic route enables efficient use of erythritol in zoonotic *Brucella*

Leticia Lázaro-Antón, Maria Veiga-da-Cunha, Aitor Elizalde-Bielsa, Nathalie Chevalier, Raquel Conde-Álvarez, Maite Iriarte, Jean Jacques Letesson, Ignacio Moriyón, Emile Van Schaftingen and Amaia Zúñiga-Ripa*

*** Correspondence:** Amaia Zúñiga-Ripa: azuniga@unav.es

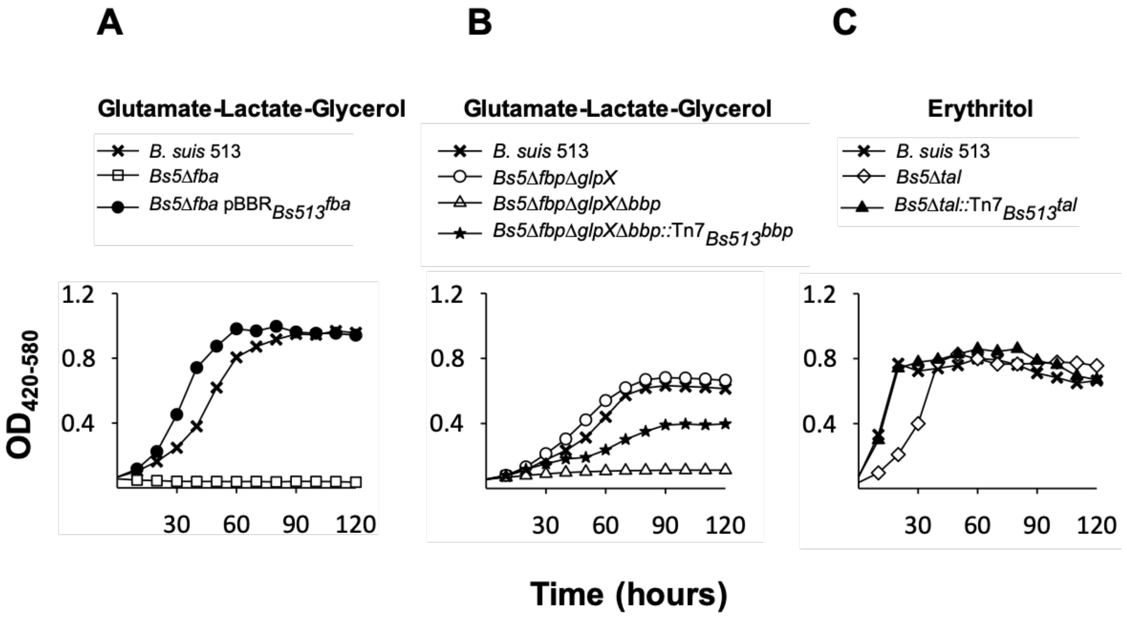

Supplementary Figure 1. (A) Growth curves in Glutamate-Lactate-Glycerol of *B. suis* 513, *Bs5*Δ*fba* and *Bs5*Δ*fba* pBBR$_{Bs513}$*fba* (carrying the complete *fba* gene). (B) Growth curves in Glutamate-Lactate-Glycerol of *B. suis* 513, *Bs5*Δ*fbp*Δ*glpX*, *Bs5*Δ*fbp*Δ*glpX*Δ*bbp* and *Bs5*Δ*fbp*Δ*glpX*Δ*bbp*::Tn7$_{Bs513}$*bbp* (carrying the complete *bbp* gene). (C) Growth curves in Erythritol of *B. suis* 513, *Bs5*Δ*tal* and *Bs5*Δ*tal*::Tn7$_{Bs513}$*tal* (carrying the complete *tal* gene). Each point represents the mean ± standard error (error bars are within the size of the symbols) of technical triplicates. The experiment was repeated at least three times with similar results.

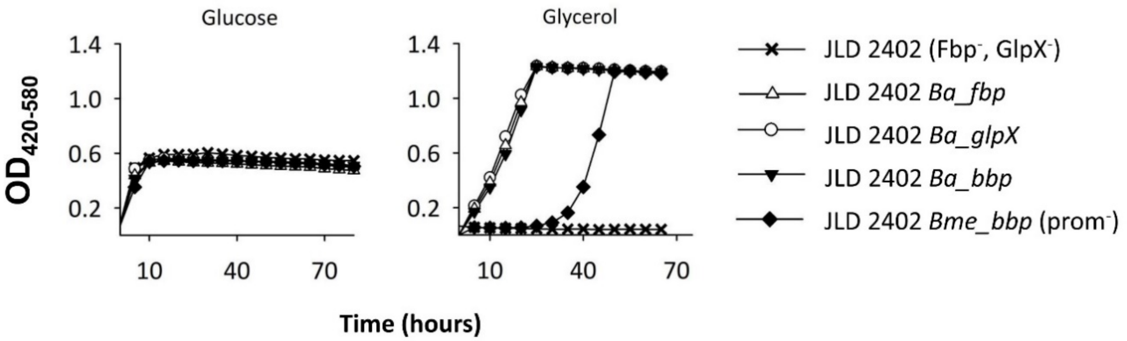

Supplementary Figure 2. *B. abortus* 2308 Fbp, GlpX and Bbp, and *B. melitensis* Bbp individually restore gluconeogenesis in *E. coli* JLD2402 (defective of Fbp and GlpX). Each point represents the mean ± standard error (error bars are within the size of the symbols) of technical triplicates. The experiments were repeated at least three times with similar results.

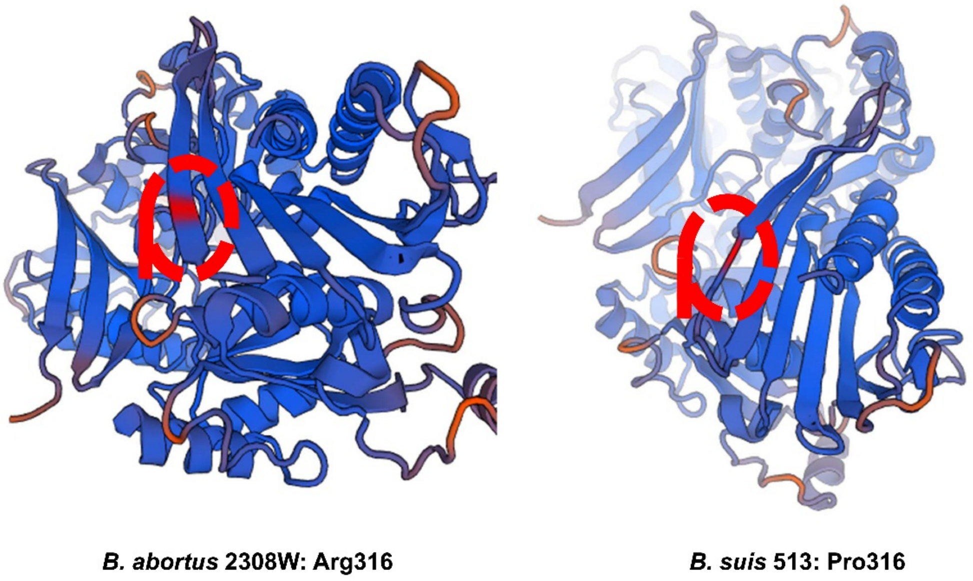

Supplementary Figure 3. The Arg316Pro mutation in *B. suis* 513 GlpX introduces a change in the predicted ß-strand structure. The models were obtained with the Swiss-Model tool (https://swissmodel.expasy.org/interactive).

Supplementary Table 1. Bacterial Strains

Strain	Characteristics[1]	Reference
Brucella		
B. suis 513	Wild type, virulent, biovar 5, smooth LPS	(1)
Bs5Δfba	*B. suis* 513 $fba_{\Delta 20\text{-}338}$	This work
Bs5Δfba pBBR$_{Bs513}$*fba*	*Bs5Δfba* carrying pBBR$_{Bs513}$*fba*	This work[2]
Bs5ΔfbpΔglpX	*B. suis* 513 $fbp_{\Delta 15\text{-}326}glpX_{\Delta 21\text{-}205}$	(2)
Bs5Δbbp	*B. suis* 513 $bbp_{\Delta 23\text{-}188}$	This work
Bs5ΔfbpΔglpXΔbbp	*B. suis* 513 $fbp_{\Delta 15\text{-}326}$ $glpX_{\Delta 21\text{-}205}$ $bbp_{\Delta 23\text{-}188}$	This work
Bs5ΔfbpΔglpXΔbbp::Tn7$_{Bs513}$*bbp*	*Bs5ΔfbpΔglpXΔbbp* carrying Tn7$_{Bs513}$*bbp*	This work[3]
Bs5Δtal	*B. suis* 513 $tal_{\Delta 23\text{-}188}$	This work
Bs5Δtal::Tn7$_{Bs513}$*tal*	*Bs5Δtal* carrying Tn7$_{Bs513}$*tal*	This work[4]
Bs5ΔfbpΔglpXΔtal	*B. suis* 513 $fbp_{\Delta 15\text{-}326}glpX_{\Delta 21\text{-}205}tal_{\Delta 23\text{-}188}$	This work
Bs5ΔfbaΔtal	*B. suis* 513 $fba_{\Delta 20\text{-}338}tal_{\Delta 23\text{-}188}$	This work
Escherichia coli		
JLD2402	TL524 *glpX*::Spc^r Δfbp_{287} $zjg\text{-}_{920}$::Tn10	(3)
JLD2402 *Ba_fbp*	JLD2402 carrying pBBR$_{BAB}$*fbp*	This work[5]
JLD2402 *Ba_glpX*	JLD2402 carrying pBBR$_{BAB}$*glpX*	This work[6]
JLD2402 *Ba_bbp*	JLD2402 carrying pBBR$_{BAB}$*bbp*	This work[7]
JLD2402 *Bme_bbp*	JLD2402 carrying pRH002$_{BME}$*bbp*	This work[8]
Stellar	F–, endA1, supE44, thi-1, recA1, relA1, gyrA96, phoA, Φ80d lacZΔ M15, Δ(lacZYA-argF) U169, Δ(mrr-hsdRMS-mcrBC), ΔmcrA, λ–	Clontech
S17λpir	Tpr Smr recA thi hsdRM+, lambda pyr phage lysogen RP4::2-Tc::Mu::Km Tn7	(4–6)
BL21(DE3)	F– ompT hsdSB (rB– mB–) gal dcm (DE3)	Novagen
PIR1	F-Δlac169 rpoS(Am) robA1 creC510 hsdR514 endA recA1 uidA(ΔMluI)::pir-116	Invitrogen
SM10λpir (pTNS2)	pTNS2 is a helper plasmid encoding the TNsABC+D transposition pathway (Amp^R)	(7)
HB101 (pRK2013)	pRK2013 is a helper plasmid for mobilization of non-self-transmissible plasmids (Km^R)	(7)

[1] Amp, ampicillin; Km, kanamycin; Nal, nalidixic acid.

[2] To obtain Bs5Δ*fba* pBBR$_{Bs513}$*fba* complemented strain, a plasmid carrying *fba* was constructed using the Gateway cloning Technology (Invitrogen). For that, a 1574 bp fragment including the complete ORF sequence was obtained from *B. suis* 513 with primers Fba-GW-F (5’-GGGGACAAGTTTGTACAAAAAAGCAGGCTTCGCGGCCTGTTTTCTATGTG -3’) and Fba-GW-R (5’-GGGGACCACTTTGTACAAGAAAGCTGGGTCCGGAAGTGGCAAAGACCAT -3’) and cloned into vector pDONR201 to obtain plasmid pLLA-22. Then, this fragment was subcloned into vector pRH002 (8) to produce plasmid pLLA-23. This plasmid was introduced into *Bs5Δfba* by mating with *E. coli* S17λpir, and the conjugants harboring this plasmid (*Bs5Δfba* pBBR$_{Bs513}$*fba*) were selected on TSA-Pmx-Cm.

[3] To obtain *Bs5ΔfbpΔglpXΔbbp*::Tn7$_{Bs513}$*bbp* complemented strain, *bbp* and its promoter were inserted into the chromosome of *Bs5ΔfbpΔglpXΔbbp* using a miniTn7 transposon (7,9). For that, a 906 bp fragment including the promotor and the complete ORF sequence was obtained from *B. suis* 513 with primers Bbp-Tn7-F (5’-CCGGGCTGCAGGAATTCTATATCCTGAAGTGGAACGA-3’) and Bbp-Tn7-R (5’-AGCTTCTCGAGGAATTTCAAACCCATTCAAGCTTACC-3’). This fragment was cloned into the linearized vector (*EcoR*I) pUC18R6KT-mini-Tn7T using the In-Fusion HD Enzyme Premix kit (Clontech) to generate plasmid pAZI-39. The plasmid was sequenced, transformed into *E. coli* PIR1 and then into S17λpir and transferred to *Bs5ΔfbpΔglpXΔbbp* by tetra-parental conjugation with *E. coli* S17λpir (carrying pAZI-39), SM10λpir (carrying pTNS2) and HB101 (carrying pRK2013). The resulting *Bs5ΔfbpΔglpXΔbbp*::Tn7$_{Bs513}$*bbp* were selected onto TSA-Pmx-Km plates that were incubated at 37ºC for 4 days. The miniTn7 insertion between genes *glmS* and *recG* was confirmed by PCR with primers Glms_B

(5’-GTCCTTATGGGAACGGACGT-3’) and PTn7-R (5’- CACAGCATAACTGGACTGATT-3’) to prove that the transposon was inserted immediately after *glmS*; and with RecG-R (5’-TATATTCTGGCGAGCGATCC-3’) and PTn7-L (5’-ATTAGCTTACGACGCTACACCC-3’) to confirm that the insertion occurred before *recG*.

[4] To obtain *Bs5*Δ*tal*::Tn7$_{Bs513}$*tal* complemented strain, a 790 bp fragment including the promotor and the complete ORF sequence was obtained from *B. suis* 513 with primers Tal(p)_Tn7_F (5’-CCGGGCTGCAGGAATTTGCAATATCGTGCGAATCTTT-3’) and Tal_Tn7_R (5’-AGCTTCTCGAGGAATTTCAGGCGATCTTCTGGCC-3’). This fragment was cloned into the linearized vector (*EcoR*I) pUC18R6KT-mini-Tn7T using the In-Fusion HD Enzyme Premix kit (Clontech) to generate plasmid pAEB-01, which was transformed into *E. coli* PIR1 and then into S17λpir, sequenced, and transferred to *Bs5*Δ*tal* by tetra-parental conjugation. The conjugants carrying the miniTn7 were named *Bs5*Δ*tal*::Tn7$_{Bs513}$*tal* (proper insertion of *tal* was verified as described above).

[5] To obtain JLD2402 *Ba_fbp,* plasmid pAZI-21 (pBBR1MCS1 carrying *B. abortus* 2308W *bbp* with its promoter) previously obtained (10), was introduced into *E. coli* JDL2402 (3) by transformation.

[6] To obtain JLD2402 *Ba_glpX*, plasmid pAZI-23 (pBBR1MCS1 carrying *B. abortus* 2308W *glpX* with its promoter) previously obtained (10), was introduced into *E. coli* JDL2402 (3) by transformation.

[7] To obtain JLD2402 *Ba_bbp, B. abortus* 2308W *bbp* and the putative promoter was amplified with primers Bbp-GW-F (5’-GGGGACCACTTTGTACAAGAAAGCTGGGTCCTATATCCTGAAGTGGAACGA-3’) and Bbp-GW-R (5’-GGGGACAAGTTTGTACAAAAAAGCAGGCTTCTCAAACCCATTCAAGCTTACC-3’) and cloned into pDONR201 to obtain plasmid pAZI-40. After sequencing, attL1-attL2 fragment of pAZI-40 was cloned into the attR1-attR2 sites of pRH002 (8) to obtain plasmid pAZI-41. This plasmid was introduced into *E. coli* JDL2402 (3) by transformation.

[8] To obtain JLD2402 *Bme_bbp, B. melitensis* 16M *bbp* was subcloned from pDONR201-BMEI1511 (11) by cloning the attL1-attL2 fragment into the attR1-attR2 sites of pRH002 (8) to obtain plasmid pAZI-42. This plasmid was introduced into *E. coli* JDL2402 (3) by transformation.

References

1. Vershilova PA, Liamkin GI, Malikov VE, Dranovskaya EA, Taran IF. *Brucella* strains from mouse-like rodents in Southwestern USSR. *Int J Syst Bacteriol* (1983) 33:399–400. doi: 10.1099/00207713-33-2-399

2. Zúñiga-Ripa A, Barbier T, Lázaro-Antón L, de Miguel MJ, Conde-Álvarez R, Muñoz PM, Letesson JJ, Iriarte M, Moriyón I. The fast-growing *Brucella suis* biovar 5 depends on phosphoenolpyruvate carboxykinase and pyruvate phosphate dikinase but not on Fbp and GlpX fructose-1,6-bisphosphatases or isocitrate lyase for full virulence in laboratory models. *Front Microbiol* (2018) 9:641. doi: 10.3389/fmicb.2018.00641

3. Donahue JL, Bownas JL, Niehaus WG, Larson TJ. Purification and characterization of *glpX*-encoded Fructose 1,6-Bisphosphatase, a new enzyme of the Glycerol-3-Phosphate regulon of *Escherichia coli*. *J Bacteriol* (2000) 182:5624–5627. doi: 10.1128/JB.182.19.5624-5627.2000

4. Miller VL, Mekalanos JJ. A novel suicide vector and its use in construction of insertion mutations: Osmoregulation of outer membrane proteins and virulence determinants in *Vibrio cholerae* requires *toxR*. *J Bacteriol* (1988) 170:2575–2583.

5. Simon LD, Randolph B, Irwin N, Binkowski G. Stabilization of proteins by a bacteriophage T4 gene cloned in *Escherichia coli*. *Proc Natl Acad Sci U S A* (1983) 80:2059–62.

6. De Lorenzo V, Timmis KN. Analysis and construction of stable phenotypes in gram-negative bacteria with Tn5- and Tn10-derived minitransposons. *Methods Enzymol* (1994) 235:386–405. doi: 10.1016/0076-6879(94)35157-0

7. Choi KH, Schweizer HP. Mini-Tn7 insertion in bacteria with single attTn7 sites: example *Pseudomonas aeruginosa*. *Nat Protoc* (2006) 1:153–161. doi: 10.1038/nprot.2006.24

8. Hallez R, Letesson J-J, Vandenhaute J, de Bolle X. Gateway-based destination vectors for functional analyses of bacterial ORFeomes: Application to the min system in *Brucella abortus*. *Appl Environ Microbiol* (2007) 73:1375–1379. doi: 10.1128/AEM.01873-06

9. Martínez-Gómez E, Ståhle J, Gil-Ramírez Y, Zúñiga-Ripa A, Zaccheus M, Moriyón I, Iriarte M, Widmalm G, Conde-Álvarez R. Genomic insertion of a heterologous acetyltransferase generates a new lipopolysaccharide antigenic structure in *Brucella abortus* and *Brucella melitensis*. *Front Microbiol* (2018) 9:1092. doi: 10.3389/fmicb.2018.01092

10. Zúñiga-Ripa A, Barbier T, Conde-Álvarez R, Martínez-Gómez E, Palacios-Chaves L, Gil-Ramírez Y, Grilló MJ, Letesson J-J, Iriarte M, Moriyon I. *Brucella abortus* depends on pyruvate phosphate dikinase and malic enzyme but not on Fbp and GlpX fructose-1,6-bisphosphatases for full virulence in laboratory models. *J Bacteriol* (2014) 196:3045–3057. doi: 10.1128/JB.01663-14

11. Dricot A, Rual JF, Lamesch P, Bertin N, Dupuy D, Hao T, Lambert C, Hallez R, Delroisse JM, Vandenhaute J, et al. Generation of the *Brucella melitensis* ORFeome version 1.1. *Genome Res* (2004) 14:2201–2206. doi: 10.1101/gr.2456204

Supplementary Table 2. Vectors/Plasmids

Vector/plasmid	Characteristics[1]	Reference
pCR2.1-TOPO	Cloning vector, Km^R	Invitrogen
pJQKm	Suicide vector, Km^R, Sac^s	[1]
pBBR1MCS1	Complementation vector, Cm^R	[2]
pRH002	pBBR1-MCS1 carrying a Gateway cassette, Cm^R	[3]
pUC18R6KT-mini-Tn7T	Mini-Tn7 vector, Km^R	[4]
pDONR201	Gateway donor vector, Km^R	Invitrogen
pBBR	pBBR1-MCS1 carrying a Gateway cassette, Cm^R	[3]
pET-21a(+)	Bacterial vector for inducible expression of N-terminally T7-tagged proteins, Amp^R	Novagen
pAZI-37	559 bp of *B. abortus* 2308W chromosomal DNA containing the *fba* (BAB2_0365) deletion allele, generated by PCR and cloned into pCR2.1-TOPO	This work[2]
pAZI-38	*BamHI*/*NotI* fragment from pAZI-37 cloned into de corresponding site of pJQKm	This work[2]
pLLA-22	1574 bp of *B. suis* 513 chromosomal DNA containing *fba* (BAB2_0365) generated by PCR and cloned into pDONR201	This work
pLLA-23	attL1-attL2 fragment of pLLA-22 cloned into the attR1-attR2 sites of pRH002	This work
pLLA-21	732 bp of *B. abortus* 2308W chromosomal DNA containing *bbp* (BAB1_0448) deletion allele, generated by PCR and cloned into pJQKm	This work[3]
pAZI-21	1388 bp of *B. abortus* 2308W chromosomal DNA containing *fbp* (BAB2_0364) and its promoter cloned into pBBR1MCS1	[5]
pAZI-23	1075 bp of *B. abortus* 2308W chromosomal DNA containing glpX (BAB1_1292) and its promoter cloned into pBBR1MCS1	[5]
pAZI-40	904 bp of *B. abortus* 2308W chromosomal DNA containing *bbp* (BAB1_0448) and its promoter, generated by PCR and cloned into pDONR201	This work
pAZI-41	BamHI/XhoI fragment from pAZI-40 cloned into de corresponding site of pRH002	This work
pDONR201-BMEI1511	*B. melitensis* 16M chromosomal DNA containing the complete ORF BMEI1511 (*bbp*), generated by PCR and cloned into pDONR201	[6]
pAZI-42	attL1-attL2 fragment of pDONR201-BMEI1511 cloned into the attR1-attR2 sites of pRH002	This work
pAZI-39	906 bp of *B. suis* 513 chromosomal DNA containing *bbp* (BAB1_0448) and its promoter, generated by PCR and cloned into pUC18R6KT-mini-Tn7T	This work
pLLA-11	707 bp of *B. abortus* 2308W chromosomal DNA containing *tal* (BAB1_1813) deletion allele, generated by PCR and cloned into pCR2.1-TOPO	This work[4]
pLLA-18	*BamHI*/*XbaI* fragment from pLLA-11 cloned into de corresponding site of pJQKm	This work[4]
pAEB-01	790 bp of *B. suis* 513 chromosomal DNA containing *tal* (BAB1_1813) and its promoter, generated by PCR and cloned into pUC18R6KT-mini-Tn7T	This work
pLLA-25	1020 bp of *B. suis* 513 chromosomal DNA containing *fbp* (BAB2_0364), generated by PCR and cloned into pET-21a(+)	This work[5]
pLLA-26	588 bp of *B. suis* 513 chromosomal DNA containing *bbp* (BAB1_0448), generated by PCR and cloned into pET-21a(+)	This work[6]
pLLA-24	984 bp of *B. suis* 513 chromosomal DNA containing *glpX* (BAB1_1292), generated by PCR and cloned into pET-21a(+)	This work[7]
pMVD-1	P316R *B. suis* 513 GlpX mutant, generated from pLLA-24 by PCR site-directed-mutagenesis	This work[7]
pMVD-2	P316R and D139G *B. suis* 513 GlpX mutant (*B. abortus*-like GlpX) generated from pMVD-1 by PCR site-directed-mutagenesis	This work[7]

[1] Amp, ampicillin; Cm, chloramphenicol; Km, kanamycin; Sac, sucrose.

[2] Construction of pAZI-37 and pAZI-38 (*fba* deletion). First, two PCR fragments were generated: oligonucleotides Fba-F1 (5'-GCGGCCTGTTTTTCTATGTG-3') and Fba-R2 (5'-AACGCCGTAACCCTTTTCAG-3') amplified a 323 bp fragment including codons 1-20 of *fba*, as well as 263 bp upstream of the *fba* start codon; oligonucleotides Fba-F3 (5'-CTGAAAAGGGTTACGGCGTTGAAATGGCAAAGCGTTATCG-3') and Fba-R4 (5'-CGGAAGTGGCAAAGACCAT-3`) were used to amplify a 236 bp fragment including the last 51 bp of *fba*. These two fragments were joined by PCR using oligonucleotides Fba-F1 and Fba-R4 for amplification and the complementary regions between Fba-R2 and Fba-F3 for overlapping. The new fragment, containing the *fba* deletion allele, was cloned

into pCR2.1-TOPO (Invitrogen) to generate plasmid pAZI-37. After verification of the sequence, the insert was excised with *BamH*I–*Xba*I and cloned into the pJQKm suicide vector to obtain pAZI-38.

[3] Construction of pLLA-21 (*bbp* deletion). Two PCR fragments were generated: oligonucleotides Bbp-F1 (5'-TGGCGGCCGCTCTAGGCATTGGTGACTTCCGCTAT-3') and Bbp-R2 (5'-CTGAATACGCTGCGAGACAT-3') amplified a 398 bp fragment including codons 1-23 of *bbp* as well as 329 bp upstream of its start codon, and oligonucleotides Bbp-F3 (5'-ATGTCTCGCAGCGTATTCAGGCCAACGGTAAGCTTGAATG-3') and Bbp-R4 (5'-ATCCACTAGTTCTAGCAAAGCCCTTGGTGGAGTTA-3') amplified a 334 bp fragment including the last 27 bp of *bbp*. Both fragments were cloned into the linearized vector (*XbaI*I) pJQKm using the In-Fusion HD Enzyme Premix kit (Clontech) to obtain pLLA-21.

[4] Construction of pLLA-11 and pLLA-18 (*tal* deletion). Two PCR fragments were generated: oligonucleotides Tal-F1 (5'-CGGGCAATTGAAAACTTCTG-3') and Tal-R2 (5'-CGCAGTGTCCACGAAAAACT-3`) amplified a 397 bp fragment including codons 1-8 of *tal*, as well as 373 bp upstream of the *tal* start codon; oligonucleotides Tal-F3 (5'-AGTTTTTCGTGGACACTGCGAAGGGCCTCGAAACCTTC-3') and Tal-R4 (5'-GTCAGGAATTTGCGAACCTG-3') were used to amplify a 310 bp fragment including the last 57 bp of *tal*. These two fragments were joined by PCR using oligonucleotides Tal-F1 and Tal-R4 for amplification and the complementary regions between Tal-R2 and Tal-F3 for overlapping. The new fragment, containing the *tal* deletion allele, was cloned into pCR2.1 (Invitrogen) to generate plasmid pLLA-11 After verification of the sequence, the insert was excised with *BamH*I–*Xba*I and cloned into the pJQKm suicide vector to obtain pLLA-18.

[5] Construction of pLLA-25 (Fbp expression and purification). The complete *B. suis* 513 *fbp* was amplified using primers Fbp-pET21-F (5'-AAGGAGATATACATAATGACATTGGTAGGTAATTTTTC-3') and Fbp-pET21-R (5'-GGTGGTGGTGCTCGAATTTCACGAAATATTCGCCGAG-3') to obtain a 1020 bp fragment, that was cloned into the pET-21a *Nde*I/*Xho*I sites using the In-Fusion HD Enzyme Premix kit (Clontech).

[6] Construction of pLLA-26 (Bbp expression and purification). The complete *B. suis* 513 *bbp* was amplified using primers Bbp-pET21-F (5'-AAGGAGATATACATAATGGCTCGGGAGATCATCTA-3') and Bbp-pET21-R (5'-GGTGGTGGTGCTCGAAAACCCATTCAAGCTTACCGTT-3') to obtain a 588 bp fragment that was cloned into the pET-21a *Nde*I/*Xho*I sites following the In-Fusion technique. The GTG start codon of *B. suis* 513 *bbp* was changed to ATG in the primers to facilitate expression of the recombinant protein.

[7] Construction of pLLA-24, pMVD-1 and pMVD-2 (GlpX expression and purification). The complete *B. suis* 513 *glpX* was amplified using primers GlpX-pET21-F (5'-AAGGAGATATACATAATGGCCAAGACCGCAGAG-3') and GlpX-pET21-R (5'-GGTGGTGGTGCTCGAAGAATTTGGACAAATCCTGATG-3') and cloned into the pET-21a *Nde*I/*Xho*I sites, as described above. However, plasmid pLLA-24 did not produce a soluble product for enzymatic analysis. Since *B. suis* 513 GlpX sequence had two amino acid changes with respect to *B. abortus* 2308W GlpX, the sequence in pLLA-24 was modified in these two positions to create a GlpX identical to that in *B. abortus* 2308W by two sequential PCR site-directed mutagenesis. First, a PCR was performed using the pLLA-24 plasmid as template and primers GlpX-P316R-F (5'- CGCGAAATCAAGGCGCGCCATC-3') and GlpX-P316R-R (5'-CACCGTCTGCGACGACGAG-3') carrying the *B. abortus* 2308W GlpX sequence (Pro316Arg codon) to create pMVD-1. Then, using this pMVD-1 plasmid as template, the Gly139Asp mutation was corrected using primers GlpX-D139G-F (5'- GGCTATCCCAAGGGTGTCGTTGA-3') and GlpX-D139G-R (5'- GGGGCCGACAGCAATCTTTT-3') to obtain plasmid pMVD-2 in which codons 316 and 139 are reverted to an arginine and glycine codons, respectively.

References

1. Scupham AJ, Triplett EW. Isolation and characterization of the UDP-glucose 4′-epimerase-encoding gene, *galE*, from *Brucella abortus* 2308. *Gene* (1997) 202:53–59. doi: 10.1016/S0378-1119(97)00453-8

2. Kovach ME, Elzer PH, Hill DS, Robertson GT, Farris MA, Roop RM, Peterson KM. Four new derivatives of the broad-host-range cloning vector pBBR1MCS, carrying different antibiotic-resistance cassettes. *Gene* (1995) 166:175–6. doi: 10.1016/0378-1119(95)00584-1

3. Hallez R, Letesson J-J, Vandenhaute J, de Bolle X. Gateway-based destination vectors for functional analyses of bacterial ORFeomes: Application to the min system in *Brucella abortus*. *Appl Environ Microbiol* (2007) 73:1375–1379. doi: 10.1128/AEM.01873-06

4. Llobet E, March C, Gimenez P, Bengoechea JA. *Klebsiella pneumoniae* OmpA confers resistance to antimicrobial peptides. *Antimicrob Agents Chemother* (2009) 53:298–302. doi: 10.1128/AAC.00657-08

5. Zúñiga-Ripa A, Barbier T, Conde-Álvarez R, Martínez-Gómez E, Palacios-Chaves L, Gil-Ramírez Y, Grilló MJ, Letesson J-J, Iriarte M, Moriyon I. *Brucella abortus* depends on pyruvate phosphate dikinase and malic enzyme but not on Fbp and GlpX fructose-1,6-bisphosphatases for full virulence in laboratory models. *J Bacteriol* (2014) 196:3045–3057. doi: 10.1128/JB.01663-14

6. Dricot A. Generation of the *Brucella melitensis* ORFeome Version 1.1. *Genome Res* (2004) 14:2201–2206. doi: 10.1101/gr.2456204

Supplementary Table 3. Chemically defined media

Components	Medium[1]	
	Gerhardt [1]	Modified Plommet[2] [2]
Carbon source		
Glucose	-	2.0 g
Erythritol		2.0 g
Glycerol	30.0 g	-
Lactic acid	5.0 g	2.0 g
Glutamic acid	1.5 g	2.0 g
Vitamins		
Thiamine. HCl	0.2 mg	0.2 g
Nicotinic acid	0.2 mg	0.2 g
Pantothenic acid	0.04 mg	0.07 g
Biotin	0.0001 mg	0.1 mg
Minerals		
$(NH_4)_2SO_4$	-	0.5 g[3]
K_2HPO_4	10.0 g	9.2 g
KH_2PO_4	-	3.0 g
$Na_2S_2O_3$	0.1 g	0.1 g
$MgSO_4$	10.0 mg	10.0 mg
$MnSO_4$	0.1 mg	0.1 mg
$FeSO_4$	0.1 mg	0.1 mg
NaCl	7.5 g	5.0 g
pH	7.0	7.0

[1] Amounts per liter.

[2] Only one of the indicated C sources but for glutamate-lactate medium where both substrates were added together.

[3] $(NH_4)_2SO_4$ was not added when glutamic acid was used as C source.

References

1. Gerhardt P, Wilson JB. The nutrition of *Brucellae*: growth in simple chemically defined media. *J Bacteriol* (1948) 56:17–24. doi: 10.1128/jb.56.1.17-24.1948

2. Plommet M. Minimal requirements for growth of *Brucella suis* and other *Brucella* species. *Zentralblatt für Bakteriologie* (1991) 275:436–450. doi: 10.1016/S0934-8840(11)80165-9
